# Accurate de novo design of heterochiral protein–protein interactions

**DOI:** 10.1038/s41422-024-01014-2

**Published:** 2024-08-14

**Authors:** Ke Sun, Sicong Li, Bowen Zheng, Yanlei Zhu, Tongyue Wang, Mingfu Liang, Yue Yao, Kairan Zhang, Jizhong Zhang, Hongyong Li, Dongyang Han, Jishen Zheng, Brian Coventry, Longxing Cao, David Baker, Lei Liu, Peilong Lu

**Affiliations:** 1 College of Life Sciences, Zhejiang University, Hangzhou, Zhejiang China; 2 Westlake Laboratory of Life Sciences and Biomedicine, Hangzhou, Zhejiang China; 3 Key Laboratory of Structural Biology of Zhejiang Province, School of Life Sciences and Research Center for Industries of the Future, Westlake University, Hangzhou, Zhejiang China; 4 Institute of Biology, Westlake Institute for Advanced Study, Hangzhou, Zhejiang China; 5 Tsinghua-Peking Joint Center for Life Sciences, Ministry of Education Key Laboratory of Bioorganic Phosphorus Chemistry and Chemical Biology, Center for Synthetic and Systems Biology, Department of Chemistry, Tsinghua University, Beijing, China; 6 Hefei National Laboratory for Physical Sciences at the Microscale, School of Life Sciences, University of Science and Technology of China, Hefei, Anhui China; 7 Department of Biochemistry, University of Washington, Seattle, WA USA; 8 Institute for Protein Design, University of Washington, Seattle, WA USA

**Keywords:** Bioinformatics, Protein folding, X-ray crystallography

## Abstract

Abiotic d-proteins that selectively bind to natural l-proteins have gained significant biotechnological interest. However, the underlying structural principles governing such heterochiral protein–protein interactions remain largely unknown. In this study, we present the de novo design of d-proteins consisting of 50–65 residues, aiming to target specific surface regions of l-proteins or l-peptides. Our designer d-protein binders exhibit nanomolar affinity toward an artificial l-peptide, as well as two naturally occurring proteins of therapeutic significance: the D5 domain of human tropomyosin receptor kinase A (TrkA) and human interleukin-6 (IL-6). Notably, these d-protein binders demonstrate high enantiomeric specificity and target specificity. In cell-based experiments, designer d-protein binders effectively inhibited the downstream signaling of TrkA and IL-6 with high potency. Moreover, these binders exhibited remarkable thermal stability and resistance to protease degradation. Crystal structure of the designed heterochiral d-protein–l-peptide complex, obtained at a resolution of 2.0 Å, closely resembled the design model, indicating that the computational method employed is highly accurate. Furthermore, the crystal structure provides valuable information regarding the interactions between helical l-peptides and d-proteins, particularly elucidating a novel mode of heterochiral helix–helix interactions. Leveraging the design of d-proteins specifically targeting l-peptides or l-proteins opens up avenues for systematic exploration of the mirror-image protein universe, paving the way for a diverse range of applications.

## Introduction

d-proteins are protein molecules whose polypeptide chains consist of d-amino acids and the achiral amino acid glycine. d-proteins, which can form specific heterochiral protein–protein interactions with natural l-protein targets, possess remarkable potential as molecular tools, therapeutics, and diagnostics due to their high bioorthogonality and stability.^1–3^ To identify d-proteins capable of binding to a target l-protein, mirror-image peptide phage display methods have been developed.^4–6^ However, it remains challenging to precisely target a specific surface region of the target protein and confirm the presence of valid binders within the initial random library.

Compared to conventional selection approaches, computational methods offer a more advanced strategy for identifying binders by targeting specific regions on the surface of a target protein. l-proteins have been designed to bind naturally existing target proteins.^7–14^ However, a significant challenge in the design of d-proteins that target l-proteins arises from our limited understanding of the heterochiral interactions between l- and d-proteins — there are only a few high-resolution 3D structures available for heterochiral protein complexes in the protein data bank (PDB).^2,15–17^ Single-helix d-peptides that bind l-protein targets^18–20^ have been generated, however, this method relies on structure information derived from a known α-helix in the l-configuration bound to the target protein, and is restricted to the one-helix scaffold. Thus far, the designed binding modes for these designer d-peptide and l-protein target complexes have not been validated with high-resolution structures. The accurate design of d-proteins that target specific surface regions of any target protein or peptide, and the de novo design of d-protein binders solely from the target protein structure, remain unsolved problems.^4,18–21^

## Results

### The mirror-image design approach

**Fig. 1:**
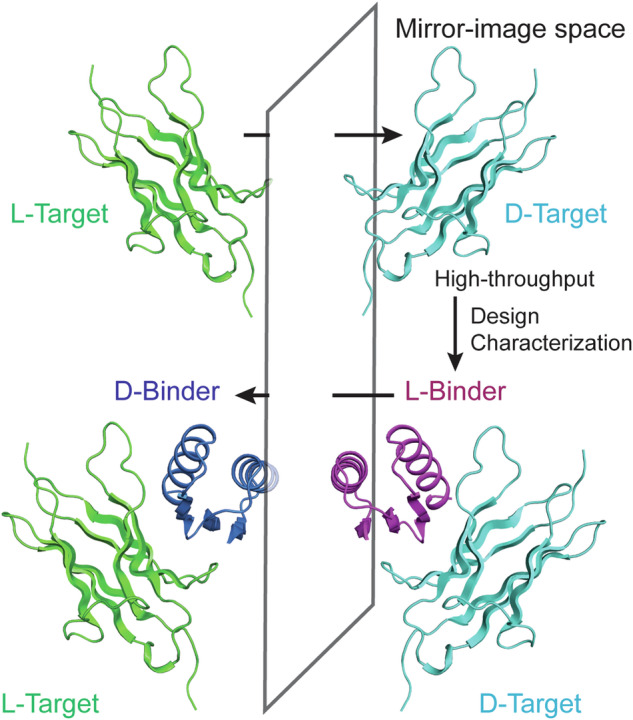
Schematic diagram of the mirror-image design approach. d-protein binders (d-Binder, blue) that bind to a selected surface region of the l-protein target (l-Target, green) can be obtained through the high-throughput design and characterization of l-protein binders (l-Binder, purple) that bind to the d-protein target (d-Target, cyan) in mirror-image space. Here, we illustrate the creation of mini d-protein binders that target the D5 domain of human TrkA (PDB ID: 1WWW).

**Fig. 2:**
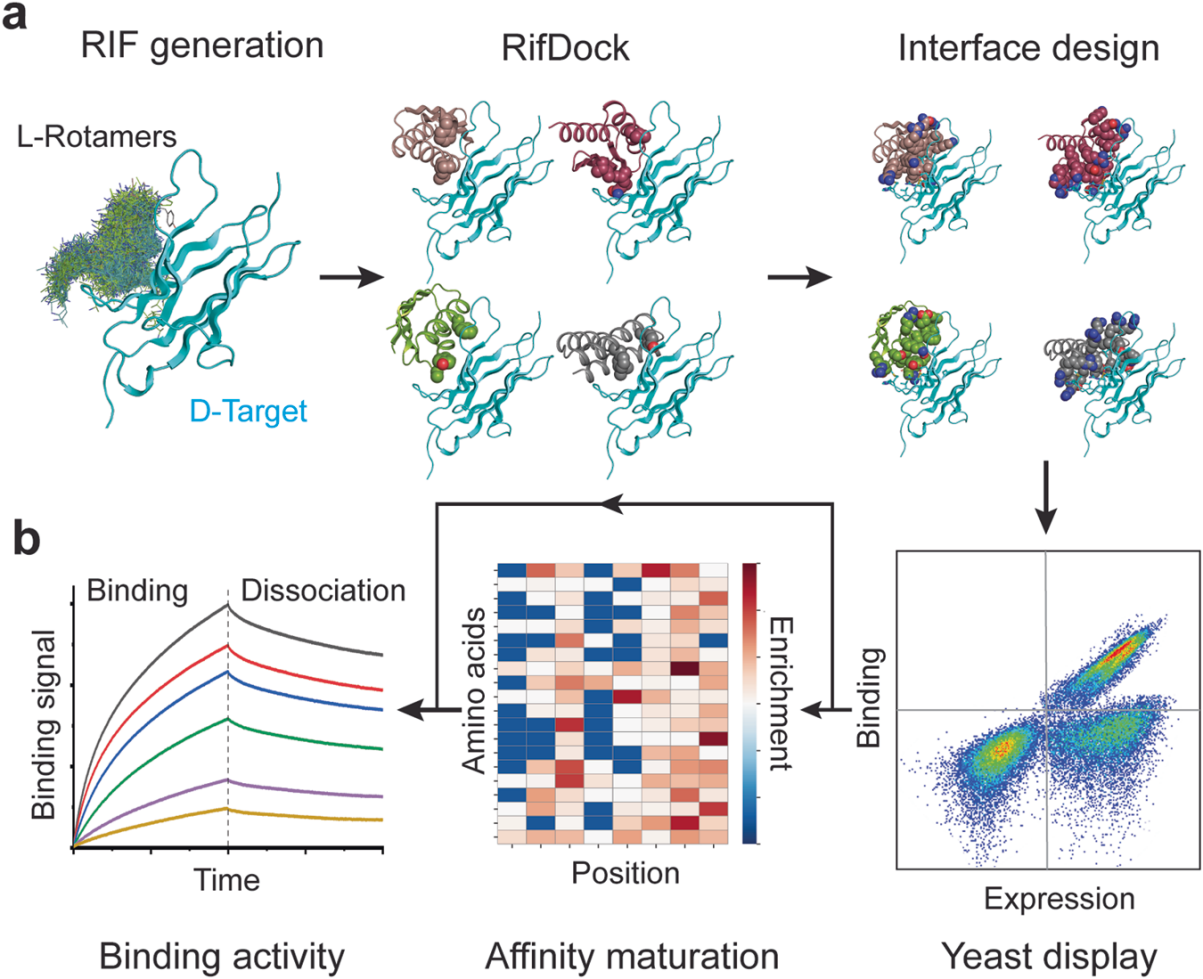
De novo design of l-protein-targeting d-proteins using an integrated computational and experimental method. **a** Key steps in the de novo design method. Pre-generated l-rotamers that interact with the selected surface region of the d-protein target are shown in lines (left panel). Mini-protein scaffolds docked on the d-protein target are shown in cartoon, with the target-interacting side chains highlighted in spheres (middle panel). Positions on the mini-protein at the interface are further designed for optimized binding (shown in spheres, right panel). **b** High-throughput experimental validation, integrating yeast display (right), directed evolution (middle) and binding affinity measurement (left). The loop arrow indicates that it is possible to obtain high-affinity binders directly from a designer library, without the need for any affinity maturation process. All structural images were generated by using PyMOL.^63^

We developed an integrated computational and experimental method that enables the design and evaluation of de novo d-protein binders for any given l-protein target (Fig. 1). In this method, the computational design, high-throughput testing, characterization, and directed evolution of the potential d-protein binders are initially performed in the mirror-image space: the natural l-protein target is chemically synthesized as the mirror image d-protein molecule; potential l-protein binders are computationally designed to interact with the d-protein form of the target molecule (Fig. 2a). The designed l-protein binders are experimentally evaluated by sorting yeast libraries displaying the designs against the refolded d-protein form of the target molecule; identified l-protein binders are expressed in *Escherichia coli*, purified and characterized in solution; the affinity of l-protein binders may be optimized by using directed evolution methods (Fig. 2b). Compared to the alternative direct design, synthesis and testing of d-protein binders against l-protein targets, this mirror-image strategy has the advantage of higher throughput by using yeast display to evaluate the designs as l-proteins in a massively parallel manner. Finally, the d-protein forms of these experimentally selected binders are chemically synthesized and characterized and will bind to the natural l-protein target for reasons of symmetry.

We hypothesize that heterochiral protein interactions likely adhere to the same principles of physical chemistry as interactions between l-proteins. These principles involve maximizing the interface through achieving chemical and shape complementarity. However, at the level of secondary structure, the geometry of the interaction must differ substantially due to chirality. We managed to calculate the computational binding energy and interface metrics for the heterochiral protein–protein or protein–peptide complexes available in the PDB^15,16,22–24^ by using Rosetta^25,26^ and found the computed values to be nearly within the range observed for l-protein complexes (Supplementary information, Fig. S1a–d), suggesting that these metrics could be applied for the in silico design and selection of d-protein binders.

We began by digitally inverting the l-protein target structure to generate the d-protein target structure. Then we used RifDock^27^ to generate the rotamer interaction field (RIF, billions of scored interacting residues) by docking discrete l-amino acids against the selected surface regions of the d-protein target structure (Fig. 2a). Our protocol was found to be effective in recovering most of the interacting l-amino acids from the d-protein target structure in known heterochiral protein complexes (Supplementary information, Fig. S1e, f). This finding demonstrates the capability of our algorithm to generate meaningful heterochiral interactions. Then, 9606 miniprotein scaffolds in the l-configuration across 5 different topologies^7^ were docked against the d-protein target guided by RIF. Interface design was performed, and more backbone geometries and interface compositions of the designer binders were sampled by using the MotifGraft algorithm^28,29^ for optimal binding.

### Massively parallel design and characterization of binders in the mirror-image space

**Fig. 3:**
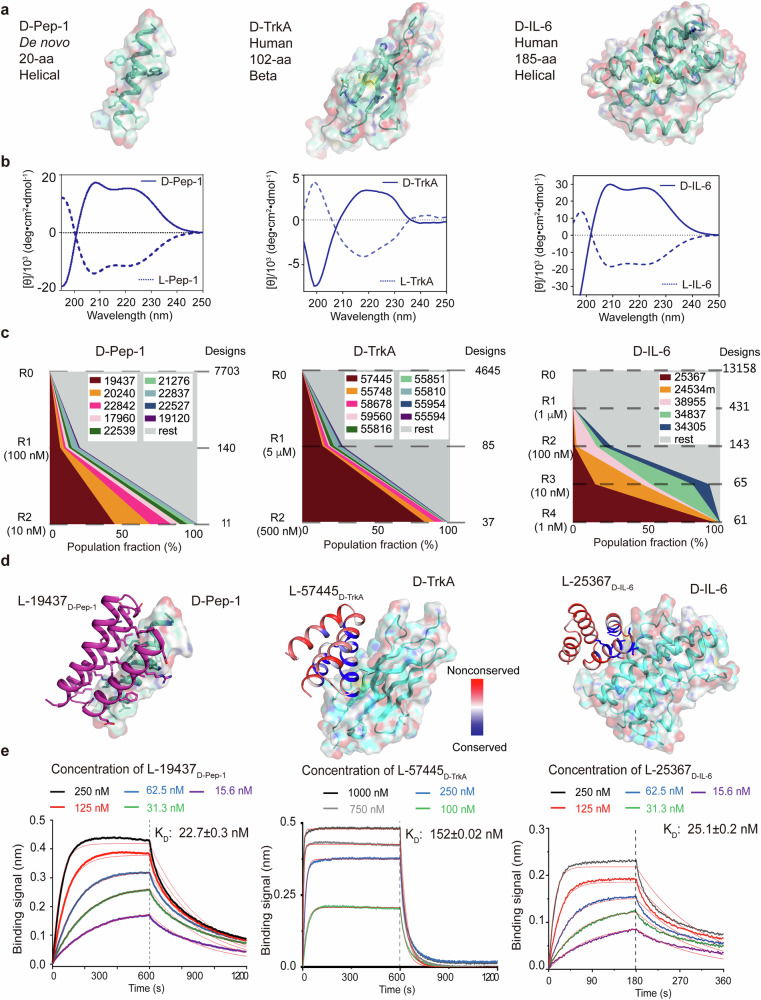
Design and evaluation of l-protein binders to mirror images of the l-protein targets. **a** d-protein target structures shown in surface and cartoon representation with targeted residues highlighted in sticks. The d-protein structure models were generated using available structures found in the PDB (1WWW^31^ for TrkA and 4O9H for IL-6). The d-Pep-1 model was generated using Rosetta. **b** CD spectra of the d-protein targets (solid lines) and l-protein targets (dashed lines). **c** High-throughput evaluation of binding. Yeasts displaying l-protein binders bound to the d-protein target are enriched during several rounds of selection at different target concentrations. The total number of designs identified in the NGS results after each round of sorting is indicated by the numbers on the right. The population fraction of each of the final surviving designs (with a population fraction larger than 0.05% in the last round of selection) in each round of selection is shown as colored schemes. **d** Computational design models of the complexes between the d-targets and the most experimentally enriched l-protein binders from panel **c**. Designer binding proteins are shown in cartoons with interface residues shown in sticks. The conserved and non-conserved positions in l-57445_d-TrkA_ and l-25367_d-IL-6_ identified in a site saturation mutagenesis experiment are colored blue and red, respectively. Consistent with the computational models, the interface residues are conserved. **e** Interaction analysis of the l-protein binders bound with d-targets by using biolayer interferometry. Please refer to Supplementary information, Tables S2–S4 for the fitted parameters.

**Fig. 4:**
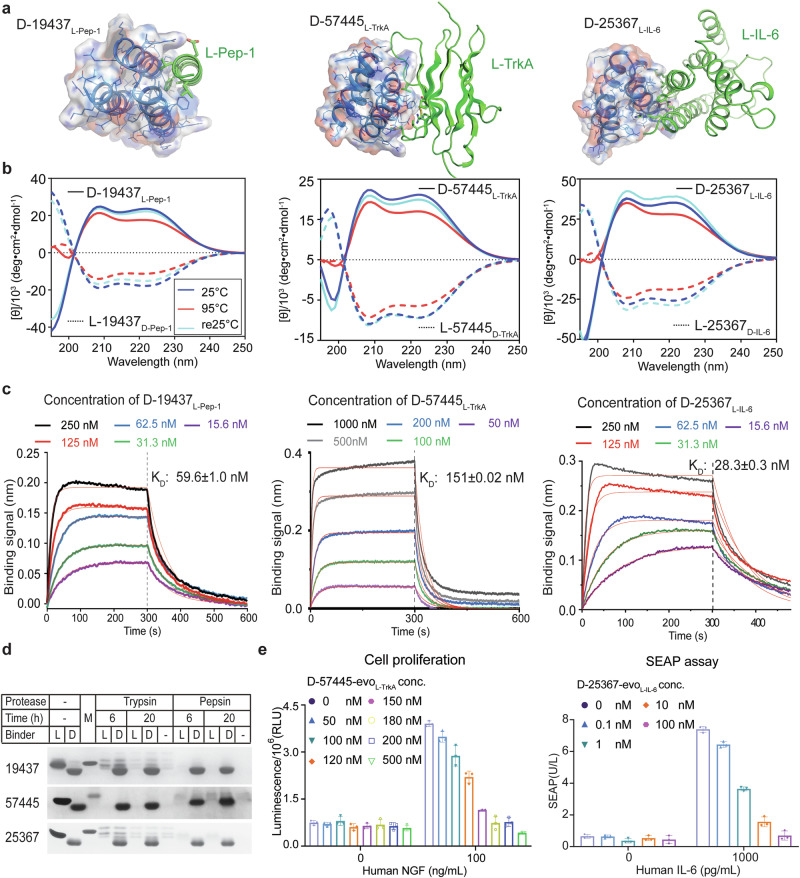
Evaluation of d-protein binders. **a** Design models of the complexes between d-protein binders and l-protein targets. l-protein target structures are shown in cartoon (green) with interface residues highlighted in sticks. Designer d-protein binders are shown in surface and cartoon representation with amino acid side chains shown in lines. **b** CD spectra of the refolded d-protein binders (solid lines) and recombinantly expressed l-protein binders (dashed lines). The spectra labeled re25 °C (cyan) correspond to the sample cooled back to 25 °C after a thermal melt scan up to 95 °C (red). No obvious unfolding transitions were detected. **c** Analysis of d-protein binder binding with l-protein targets by using biolayer interferometry. Please refer to Supplementary information, Tables S2–S4 for the fitted parameters. **d** Proteolysis analysis of d-protein binders and their mirror images. l-protein and d-protein binders treated with trypsin or pepsin for 6 h or 20 h were subjected to SDS-PAGE analysis and stained with Coomassie blue. M, protein marker (10 kDa). **e** Inhibition of target signaling by designer d-protein binders. Left panel, the proliferation of human TF-1 cells stimulated by the native NGF-TrkA signaling pathway was inhibited by d-57445-evo_l-TrkA_. Right panel, d-25367-evo_l-IL-6_ inhibits the IL-6-IL-6R signaling in a cell-based assay. NGF and IL-6 bind to TrkA and IL-6R, respectively, with nanomolar affinity.^31,32^ The mean values from triplicates are shown with error bars representing standard deviations.

To test our mirror-image design protocol, we used an artificial alpha helical peptide (named l-Pep-1) and two natural l-proteins of pharmacological significance, which are the D5 domain of human tropomyosin receptor kinase A (residues 283–384, hereafter referred to as l-TrkA) and the human interleukin-6 (residues 28-212, l-IL-6). These peptide and protein targets (hereafter referred to as protein targets for simplicity) have different origins, shapes and surface characteristics (Figs. 3a, 4a). We designed l-Pep-1 as an amphipathic alpha-helix with arbitrary hydrophobic residues and hydrophilic residues on its nonpolar and polar faces, respectively (Supplementary information, Fig. S2a). The design model was nearly identical to the structure predicted by AlphaFold^30^ (Supplementary information, Fig. S2b). l-TrkA and l-IL-6 bind nerve growth factor (NGF)^31^ and IL-6 receptors (IL-6R),^32^ respectively, and are involved in a variety of signaling processes. There are no known examples of d-proteins that bind l-Pep-1, l-TrkA or l-IL-6.

We chemically synthesized the mirror-image configuration of the l-protein targets, namely d-Pep-1, d-TrkA, and d-IL-6, on a milligram scale. All these targets were labeled with an N-terminal biotin (Supplementary information, Figs. S3–S5). d-Pep-1 was soluble in water solution. Synthesized d-TrkA and d-IL-6 were dissolved in 8 M urea and 6 M guanidinium chloride (GdmCl) solution, respectively, and then folded by dialysis against buffer without denaturants. Folded d-TrkA and d-IL-6 were further purified by size-exclusion chromatography (SEC) (Supplementary information, Fig. S2c, d). The circular dichroism (CD) spectra of the three d-proteins were of opposite sign to those measured for the folded l-protein targets (Fig. 3b).

For d-Pep-1, we selected the hydrophobic region for the binders to target. We digitally inverted the chirality of the TrkA (PDB ID: 1WWW^31^) and IL-6 (PDB ID: 4O9H) structures to generate the d-TrkA and d-IL-6 structures. For d-TrkA and d-IL-6, we designed against the cognate regions in l-TrkA and l-IL-6 that interact with NGF and IL-6R, respectively, for potential blockage of the downstream signaling pathways. We designed and selected 4500–14,000 l-protein binders composed of 50–65 amino acids for each d-protein target, which were at least locally optimal in design calculations sampling from an estimated sequence space on the order of 10^20^ (~10,000 protein backbones × 10^16^ interface residue combinations). Specifically, there were 7703 binders generated for Pep-1, 13,158 binders for IL-6, and 4642 binders for TrkA. The computational interface metrics for these designer heterochiral protein complexes were at similar levels to those for heterochiral or homochiral protein complex structures available in the PDB (Supplementary information, Fig. S1a–d). Pools of oligonucleotides encoding the designer l-protein mini-binders were synthesized, amplified, and cloned into a yeast surface-expression vector for display. Yeast cells displaying designs that bound the d-protein target were enriched by 2–4 rounds of fluorescence-activated cell sorting (FACS) (Fig. 3c). The frequency of each design in the initial yeast libraries and the sorted cell populations was determined by the next-generation sequencing (NGS). The top enriched binders for each target have different sequences (Supplementary information, Fig. S6).

We expressed and purified the most highly enriched l-protein binders for each of the d-targets in *E. coli* and characterized their binding affinity with the d-protein targets (Fig. 3d, e). The binder l-19437_d-Pep-1_ showed a binding affinity of 22 nM to d-Pep-1, as measured using biolayer interferometry. Additionally, the binder l-57445_d-TrkA_ exhibited an affinity of 152 nM towards d-TrkA. Lastly, the binder l-25367_d-IL-6_ bound to d-IL-6 with an affinity of 25 nM. The binding of l-19437_d-Pep-1_, l-57445_d-TrkA_, and l-25367_d-IL-6_ to d-targets was significantly diminished or eliminated upon mutation of essential designer interface residues, consistent with the design model (Supplementary information, Fig. S7).

The binding affinity of l-protein binders were optimized by using directed evolution methods. We constructed a site saturation mutagenesis (SSM) library for l-57445_d-TrkA_ and l-25367_d-IL-6_ and identified mutations that increased binding. We systematically generated combinatorial mutation and random mutagenesis libraries to identify protein binders capable of binding to d-TrkA or d-IL-6 with high affinity. Two binders were successfully identified from these libraries, namely l-57445-evo_d-TrkA_ and l-25367-evo_d-IL-6_ (Supplementary information, Fig. S6d). The affinity between l-57445-evo_d-TrkA_ and d-TrkA was measured to be 7.8 nM using biolayer interferometry, whereas the affinity between l-25367-evo_d-IL-6_ and d-IL-6 was measured to be 0.7 nM (Supplementary information, Fig. S8a, b, f, g).

### Characterizing the designer d-protein binder proteins

The d-protein forms of the well-characterized l-protein binders were chemically synthesized, folded and characterized (Fig. 4; Supplementary information, Figs. S8, S9). Due to the small size and stable nature of the proteins, protein synthesis and folding was straightforward. All d-protein binders had CD spectra that were comparable to those measured for the recombinantly expressed l-protein binders, with opposite sign (Fig. 4b). These results were in line with the design models of d-protein binders. The d-protein binders were hyper-stable in the thermo-melting experiments: the CD spectra at 95 °C are very close to those at 25 °C (Fig. 4b). Compared to the l-protein binders, the d-protein binders showed much greater resistance to protease treatment even under very acidic conditions (pH 2.5 for pepsin). All l-protein binders were completely digested by trypsin or pepsin after a 6-h incubation period, while all d-protein binders remained intact even after a 20-h incubation period (Fig. 4d).

The folded d-protein binder binds the l-protein target with an affinity close to that measured between the cognate l-protein binder and d-protein target (Fig. 4c). The binders D-19437_l-Pep-1_, d-57445_l-TrkA_ and d-25367_l-IL-6_ bound to l-Pep-1, l-TrkA and l-IL-6 with affinities of 59 nM, 151 nM and 28 nM, respectively, as measured by using biolayer interferometry. The evolved binders d-57445-evo_l-TrkA_ and d-25367-evo_l-IL-6_, exhibited affinities towards l-TrkA and l-IL-6 of 1 nM and 3 nM, respectively, as determined through biolayer interferometry (Supplementary information, Fig. S8c, h). To further validate the binding affinities, isothermal titration calorimetry (ITC) was employed for binders d-19437_l-Pep-1_ and d-25367-evo_l-IL-6_ (Supplementary information, Fig. S10), while microscale thermophoresis (MST) was used for d-57445-evo_l-TrkA_ (Supplementary information, Fig. S8d and Table S5). The results showed that d-19437_l-Pep-1_, d-57445-evo_l-TrkA_, and d-25367-evo_l-IL-6_ bound to l-Pep-1, l-TrkA, and l-IL-6 with affinities of 12 nM, 1 nM, and 89 nM, respectively. It is noteworthy that the ITC analysis suggested the possible presence of two sequential d-protein binding sites on l-IL-6, with the second binding site exhibiting a weaker affinity compared to the first binding site. The complexes between d-proteins and l-proteins were assembled in vitro, and binding of d-proteins to the corresponding l-proteins was confirmed by coelution in SEC (Supplementary information, Fig. S11).

The three designer binders, d-19437_l-Pep-1_, d-57445-evo_l-TrkA_ and d-25367_l-IL-6_, exhibit high enantiomeric and target specificity. The binding between l-protein binders and their corresponding l-protein targets was undetectable, suggesting that the designer d-protein binders have high enantiomeric specificity (Supplementary information, Fig. S12b–d). To evaluate their target specificity, we tested the interactions of the d-protein binders with all the l-protein targets. While the cross-reactivity of these d-binders is generally low, there is some binding observed between d-57445-evo _l-TrkA_ and l-Pep1 (Supplementary information, Fig. S12).

In cell-based experiments, d-57445-evo_l-TrkA_ and d-25367-evo_l-IL-6_ were shown to effectively block downstream signaling pathways by direct competing with NGF and IL-6R, respectively (Fig. 4e; Supplementary information, Fig. S8e). d-57445-evo_l-TrkA_ inhibits the phosphorylation of Akt and Erk kinases downstream of TrkA signaling and inhibits cell proliferation with an EC_50_ value of ~120 nM. Similarly, d-25367-evo_l-IL-6_ inhibits the IL-6-IL-6R signaling in a reconstituted cell-based assay with an EC_50_ value of ~1 nM. Both inhibitors demonstrated significant potency and exhibited concentration-dependent effects. These findings suggest that the designed d-protein binders for TrkA and IL-6 effectively bind to their intended regions on targets.

### Structure validation of the designer heterochiral protein complexes

**Fig. 5:**
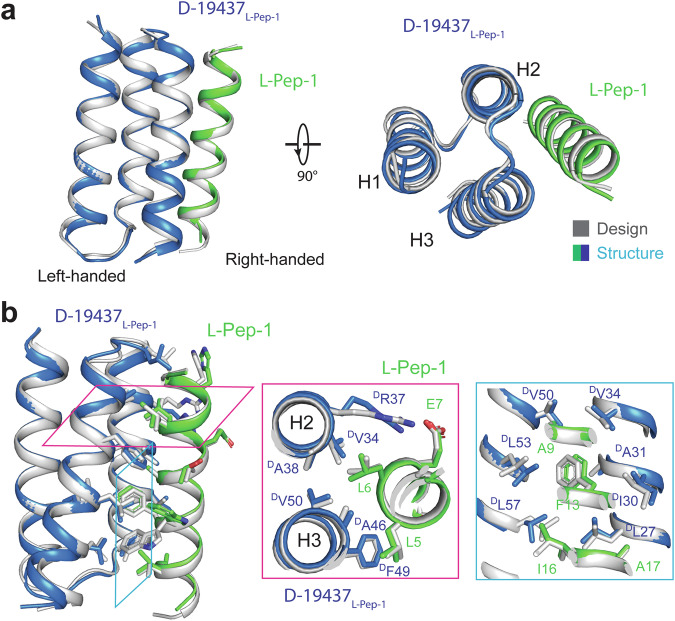
Crystal structure of the d-19437–l-Pep-1 heterochiral protein complex agrees with the design model. **a** Superposition of the crystal structure (l-Pep-1 in green; d-19437_l-Pep-1_ in blue) and the design model (gray). The crystal structure is nearly identical to the design model, with a Cα RMSD value of 0.6 Å. **b** Zoomed-in view of the heterochiral interface. The side chains of interface residues of l-Pep-1 and d-19437_l-Pep-1_ are shown in sticks. Leu6 and Phe13 of l-Pep-1 are nestled in a hydrophobic groove formed by nonpolar amino acids from d-19437_l-Pep-1_. Buttressing these hydrophobic contacts, the carboxylate side chain of Glu7 of l-Pep-1 formed charge–charge interactions with the side chain of ^D^Arg37 of d-19437_l-Pep-1_.

The l-protein binder structures predicted by AlphaFold^30^ are very similar to the design models (with root mean square deviation (RMSD) values of Cα atoms less than 1 Å) (Supplementary information, Fig. S13a–c), which strongly suggest that binders were able to fold into the designed structures. For the heterochiral complexes, we subjected the purified complexes to crystallization and determined the structure of d-19437_l-Pep-1_ in complex with l-Pep-1 at 2.0 Å resolution (Fig. 5; Supplementary information, Table S1). The designer d-19437_l-Pep-1_, consisting of three helices connected by two short loops, binds to l-Pep-1 through two helices in its crystal structure (Fig. 5a). This interaction involves the formation of numerous hydrophobic interactions, which are further enhanced by certain intermolecular electrostatic interactions (Fig. 5b). The crystal structure is nearly identical to the computational design model with a Cα RMSD of 0.6 Å for all aligned Cα atoms. The designed interface residues have conformations that match the design model with almost pinpoint accuracy, which showed clear electron density in an unbiased omit map (Supplementary information, Fig. S13d, e). We also determined a 2.2 Å resolution crystal structure of l-19437_d-Pep-1_ in complex with d-Pep-1, which is nearly identical to the mirror-image of the d-19437–l-Pep-1 complex structure (Supplementary information, Fig. S14 and Table S1).

### New insights into the interactions between l- and d-proteins

**Fig. 6:**
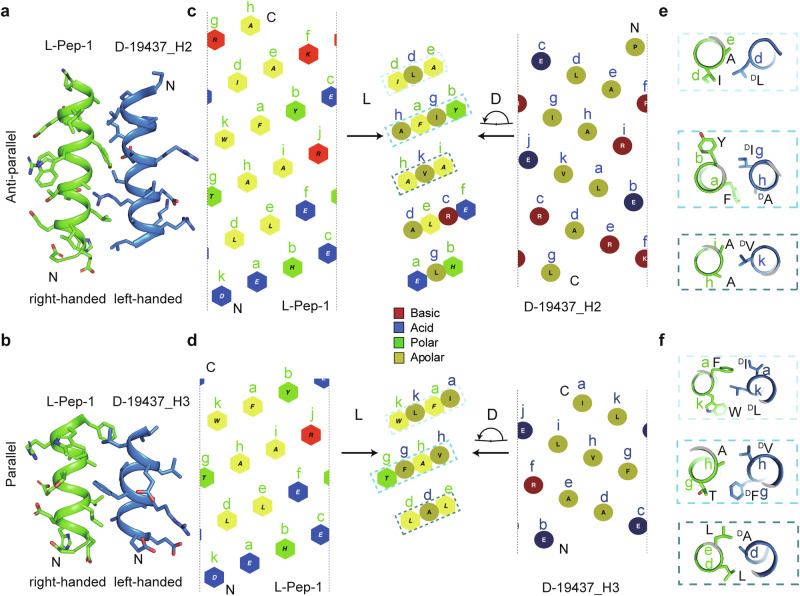
Heterochiral helix–helix interactions in the crystal structure of the d-19437–l-Pep-1 complex. **a**, **b** Heterochiral helical interfaces between l-Pep-1 and individual helix of d-19437_l-Pep-1_ (l-Pep-1 in green; helices of d-19437_l-Pep-1_ in blue. **a** H2 helix of d-19437_l-Pep-1_. **b** H3 helix of d-19437_l-Pep-1_.) **c**, **d** Helical net diagrams. Left panels, l-Pep-1. Right panels, H2 (**c**) and H3 (**d**) of d-19437_l-Pep-1_. Middle panels, heterochiral helix-helix interface. **c** Antiparallel. **d** Parallel. The hexagons and circles indicate the positions of Cα atoms from l-Pep-1 and d-19437_l-Pep-1_, respectively. The uppercase letters in hexagons and circles represent the identities of the corresponding amino acids. The lowercase letters identify the hendecad positions (abcdefghijk). Colored rectangles in dashed lines indicate the three distinct packing layers between the two heterochiral hendecad repeats. Helical net diagrams were generated by using NetWheels.^64^ **e**, **f** Heterochiral helix–helix packing arrangements in each of the three packing layers in **c** and **d**. **e** Antiparallel. **f** Parallel.

Our heterochiral complex structure provides fresh insights into the interactions between a helical l-peptide and a small d-protein binder. l-Pep-1 forms antiparallel and nearly parallel helix–helix interactions with the second (H2) and the third (H3) helices of d-19437_l-Pep-1_, respectively (Fig. 6a, b). l-Pep-1, d-19437-H2, and d-19437-H3 are nearly untwisted helices with periodicities of 3.64, 3.68, and 3.7 residues per α-helical turn, respectively, which would approximately align every 11^th^ residue along the α-helix axis after three full turns in a short range (referred to as hendecad motif^21,33^ in which residue positions are coded as abcdefghijk) (Fig. 6c, d). Both the antiparallel and parallel heterochiral helix–helix interfaces exhibit regular patterns of interaction by forming the knobs-into-holes interaction, which involves matching the shape and charge complementarity. For example, in the case of the antiparallel helix–helix interaction, a residue positioned at “e” in the l-helix inserts into a pocket formed by four residues positioned at “g, d, c, and k” in the d-helix. The periodic interaction pattern, consisting of three distinct packing layers between the two heterochiral hendecad motifs, is illustrated by overlaying the two helical net diagrams (Fig. 6c, d, the middle panel). The interface residues from the two heterochiral helices form parallel layers in space and interlock through repetitive knobs-into-holes interactions, resembling two coupled gears revolving in opposite directions. By contrast, the helix–helix interactions of homochiral untwisted parallel or antiparallel α-helices^33^ are more complex than those of heterochiral α-helices. These interactions exhibit a periodic pattern but form intersecting layers, as illustrated in the helical net diagrams (Supplementary information, Fig. S15).

To our knowledge, this is the first experimental evidence clearly demonstrating the predicted mode of interaction in a heterochiral helical protein complex, as initially proposed by Crick in 1953.^34^ Additionally, previous studies have shown that heterochiral hendecad motifs play a role in the packing of helices in racemic crystals of helical peptides.^21,35^ However, it should be noted that these studies did not measure the binding affinity between the two racemic peptides and the observed interface may be a result of the racemic crystal packing. Our results show that hendecad motifs contribute to the heterochiral helix–helix packing in a heterochiral protein complex.

## Discussion

The de novo design of d-proteins to target specific natural l-proteins poses a rigorous challenge due to the limited availability of structural information regarding such heterochiral protein complexes. However, our study demonstrates that the computational method we developed is sufficiently accurate. By successfully designing d-protein binders that effectively interact with defined surface regions of target l-proteins and by achieving agreement between the crystal structure and the design model of the d-19437–l-Pep-1 complex, we have shown that interactions between l-proteins and d-proteins can now be designed with atomic-level precision. To our knowledge, these are the first demonstration of the accurate de novo design of heterochiral protein complexes. Our mirror-image design approach, which involves initially designing and characterizing l-protein binders for the d-protein form of a natural target in a high-throughput manner and subsequently converting the most effective l-protein binders to d-protein binders through chemical synthesis, has proven to be robust and efficient.

Our analysis and results, in combination with previous studies, indicate that the protein motifs governing heterochiral protein–protein interactions, such as hendecad motifs^21^ and rippled-sheet motifs,^36^ differ significantly from those responsible for homochiral protein–protein interactions. However, similar to l-protein–l-protein interactions,^37^ d-protein binders tend to optimize the overall impact of chemical and geometric complementarity in order to bind natural l-protein targets. This is achieved by forming extensive van der Waals packing and electrostatic interactions across the heterochiral protein–protein interface. Our study represents a significant advancement towards a comprehensive understanding of the structural principles underlying heterochiral protein–protein interactions, which could ultimately enhance the success rate of protein design.

The d-protein binder molecules designed in our study were limited to a maximum of 65 amino acids, but they were easily synthesizable and folded into stable structures. Although the protein targets in our research were up to 185 residues in length, our approach has the potential to be applied to even larger protein targets, including transmembrane proteins. For example, a four-transmembrane-domain transporter can be chemically synthesized.^38^ Additionally, recent advancements in the robust, fast, and on-demand synthesis of proteins offer the possibility of efficiently producing d-protein molecules.^39^ The chemical synthesis of proteins not only allows for the incorporation of d-amino acids but also provides the opportunity to incorporate other non-canonical amino acids with unique chemical and geometric properties.

The accurate design of d-proteins targeting l-proteins forms the basis for a diverse range of applications in the development of molecular tools, therapeutics, and diagnostics. In future investigations, a variety of custom-designed proteins have the potential to facilitate mirror-image biology^40,41^ by connecting the natural realm with its mirrored counterpart. Numerous intriguing possibilities emerge, such as the development of designer l-proteins capable of catalyzing chemical reactions of mirror-image substrates, as well as d-proteins designed to recognize or modify naturally existing DNA or RNA molecules.

## Materials and methods

### Computational design

The crystal structure (PDB ID: 1WWW) of human nerve growth factor in complex with the ligand-binding domain of TrkA receptor and interleukin-6 in complex with a camelid fab fragment (PDB ID: 4O9H) were refined with the phenix.rosetta_refine program.^42^ The structure of l-Pep-1 was predicted by using AlphaFold, and 5 models were generated. The TrkA D5 domain, IL-6 and predicted model of l-Pep-1 were used for subsequent steps. All the l-protein target structures were mirror-imaged to the *y*-*z* plane by making opposite sign of the *x* coordinates to obtain the structures of the d-targets for docking and design. The design protocol is slightly modified from that for the design of the homo-chiral protein–protein interface.^7^ Briefly, Disembodied l-amino acids were docked against the desired binding surface on the d-protein target for the generation of the rotamer interaction field (RIF),^7^ with favorable hydrogen-bonding or non-polar interactions. For rapid searching of rotamers aligning with a given miniprotein scaffold, all the rotamer ensembles were stored in a six-dimensional hash table. Miniprotein scaffolds were mutated to poly-valine first and docked against the d-targets by using PatchDock,^43^ and the identified seeding positions were further refined by RifDock.^27^ Miniprotein scaffolds in the library were docked into the RIF using a branch-and-bound searching strategy to generate ~1 × 10^5^ docked models. Rosetta FastDesign protocol^26^ was used to optimize the interfaces for the docking models. During the design stage, we allowed for the design of the previously generated RIF residues. Computational metrics of all the designed models were calculated by using Rosetta. A maximum likelihood estimator is used to assess the designed models generated during the interface design stage. For the MotifGraft^9^ stage, ~1000 motifs interacting with the d-protein target were selected and miniproteins were grafted onto these motifs to generate ~1–10 million designs for every d-target. The heterochiral interfaces of the grafted models were further optimized by using Rosetta FastDesign protocol. 4642–14,000 designer binders for d-TrkA, d-IL-6 and d-Pep-1 were selected based on the computational interface metrics for experimental evaluation. Specifically, there were 7703 binders generated for Pep-1, 13,158 binders for IL-6, and 4642 binders for TrkA. During RifDock and interface design, we used the Rosetta energy function “beta”,^44,45^ which is suitable for calculating the energetics for d-amino acids in cyclized peptides.^46–50^

### Chemical synthesis

#### Materials

Rink Amide AM resin (0.27 mmol/g, 0.55 mmol/g loading) were purchased from Tianjin Nankai HECHENG S&T Co., Ltd (Tianjin, China). Fmoc-protected amino acids, Fmoc-d-protected amino acids (Fmoc-d-Ala-OH, Fmoc-d-Arg(Pbf)-OH, Fmoc-d-Asn(Trt)-OH, Fmoc-d-Asp(OtBu)-OH, Fmoc-d-Cys(Trt)-OH, Fmoc-d-Gln(Trt)-OH, Fmoc-d-Glu(OtBu)-OH, Fmoc-Gly-OH, Fmoc-d-His(Trt)-OH, Fmoc-d-Ile-OH, Fmoc-d-Leu-OH, Fmoc-d-Lys(Boc)-OH, Fmoc-d-Met-OH, Fmoc-d-Phe-OH, Fmoc-d-Pro-OH, Fmoc-d-Ser(OtBu)-OH, Fmoc-d-Thr(OtBu)-OH, Fmoc-d-Trp(Boc)-OH, Fmoc-d-Tyr(OtBu)-OH, Fmoc-d-Val-OH), Fmoc-beta-Ala-OH, Fmoc-(Dmb)Gly-OH, Fmoc-d-Glu(OAllyl)-OH, Fmoc-d-Cys(Acm)-OH and 1-Ethynyl-4-(4-pentylcyclohexyl)cyclohexanol (RBM) were purchased from Jiangsu ShenLang Biotech Co., Ltd (Nantong, China). *N*,*N*-dimethylformamide (DMF), triisopropylsilane (TIPS), trifluoroacetic acid (TFA), Anisyl sulfide, PdCl_2_, PdOAc_2_, 2,2’-[azobis(1-methylethylidene)]bis[4,5-dihydro-1H-imidazole dihydrochloride (VA-044), pentanedionen and DL-dithiothreitol (DTT) were purchased from J&K Scientific Ltd (Beijing). Dichloromethane (DCM), *N*,*N*-diisopropyl-carbodiimide (DIC), Ethyl cyanoglyoxylate-2-oxime (Oxyma), *N*,*N*-diisopropylethylamine (DIEA), Tris (2-carboxyethyl)phosphine hydrochloride (TCEP·HCl), Guanidine hydrochloride (GdmCl), methanol and sodium chloride (NaCl) were purchased from Shanghai Titan Scientific Co., Ltd. 1,2-Ethanedithiol (EDT) was purchased from TCI (Shanghai, China) Development Co., Ltd. 4-Mercaptophenylacetic acid (MPAA) was purchased from Alfa Aesar. Sodium nitrite (NaNO_2_) was purchased from Beijing Chemical Works (Beijing, China). Piperidine was purchased from Sinopharm Chemical Reagent Co., Ltd. Ethyl ether was purchased from Modern Oriental (Beijing) Technology Development Co., Ltd. Acetonitrile was purchased from Mallinckrodt Baker, Inc. Disodium hydrogen phosphate, *O*-(6-Chloro-1-hydrocibenzotriazol-1-yl)-1,1,3,3-tetramethyluroniumhexafluorophosphate (HCTU) and sodium dihydrogen phosphate was purchased from Shanghai Bidepharmatech Co., Ltd. t-butyl mercaptan was purchased from Acros Organics. Concentrated hydrochloric acid was purchased from Beijing Tongguang Fine Chemicals Company. Boc-GABA-OH was purchased from Gl Biochem (Shanghai) Ltd. Triphenylphosphine-3,3’,3”-trisulfonic acid trisodium salt (TPPTS) was purchased from Shanghai Aladdin Bio-Chem Technology Co., LTD. NaBH_4_ was purchased from Sigma-Aldrich Co.

#### Peptide segment synthesis

All peptides were made by standard Fmoc solid-phase peptide synthesis (Fmoc SPPS) and were automatically synthesized by the Liberty blue microwave peptide synthesizer (CEM Corporation). Hydrazide resin was used to prepare hydrazide terminal peptide fragments, and amide resin was used to prepare amide terminal peptide fragments. First, the Fmoc protecting group was removed at 90 °C for 1 min using a solution of 10% piperidine and 0.1 M Oxyma in DMF. The resin was then washed 3 times with DMF. The resin (0.25 mmol), 4 equivalents of (eq.) Fmoc-protected amino acids (0.2 mM, 5 mL, dissolved in DMF), 4 eq. Oxyma (1 mM, 1 mL, dissolved in DMF), and 4 eq. DIC (0.5 mM, 2 mL, dissolved in DMF) were mixed and coupled under microwave heating at 90 °C for 2 min. The standard synthesis procedure concluded with washing the resin three times with DMF. At the end of the procedure, the peptide was cleaved from the resin by treating it for 3 h with TFA cleavage cocktails (TFA/TIPS/thioanisole/water/EDT, 82.5:5:5:5:2.5, v/v/v/v/v). The TFA solution was then concentrated with nitrogen agitation, precipitated with ice-cold diethyl ether, centrifuged and then the supernatant was poured out. This process was repeated three times. The resulting precipitate, a crude peptide, was subsequently purified using RP-HPLC to obtain the pure peptide of interest.

#### RP-HPLC purification

Reversed-phase HPLC was performed on Shimadzu Prominence HPLC. A pump mobile phase was acetonitrile with 0.1% TFA, and B pump mobile phase was deionized water with 0.1% TFA. Each peptide segment and reaction purification were optimized separately for different gradients. The crude peptide was dissolved in 50% acetonitrile in water with 0.1% TFA, filtered with a 0.22-µm filter, and the purified solution is lyophilized to obtain pure peptide powder.

#### N-terminal biotin modification

Above synthetic resin with 4 eq. d-Biotin (0.5 mM, 2 mL, dissolved in DMF), 4 eq. Oxyma, 4 eq. DIC were mixed and coupled under microwave heating at 90 °C for 2 min. This process was repeated three times.

#### RBM peptide synthesis^38^

The amino acid position where the RBM group needs to be introduced was first synthesized using the microwave. The resin was then transferred to the reaction tube, where 2 eq. RBM was added and stirred for 40 min. This step was repeated twice. After the reaction, the resin was washed alternately with DMF and DCM. Next, 5 eq. NaBH_4_ (dissolved in DMF) was mixed with the resin for 10 min, repeating this step twice, followed by washing the resin. Finally, 4 eq. Fmoc-d-protected amino acids, 4 eq. Oxyma and 4 eq. DIC were mixed with the resin and left to react overnight. The resin was then washed. The resin was transferred to the microwave to continue coupling the rest of the sequence. Next, the resin was moved to the reaction tube, and SnCl_2_ (10 mg/mL, 10 μL concentrated HCl, dissolved in DMF) was added to reduce the -NO_2_ group. The Arg tag ((beta)Ala-Arg-Arg-Arg-Arg-Boc) was then coupled in the microwave. After piperidine treatment for 30 min, 10 eq. Boc-GABA-OH, 4 eq. Oxyma and 4 eq. DIC were added for overnight coupling.

#### Removal of GABA

Dissolved crude peptide fragments in 6 M GdmCl solution (pH 7). Allow the reaction to proceed at room temperature for 10 min.

#### MPAA thioester peptide synthesis^51^

Dissolved crude peptide fragments (10 mg/mL) in 6 M GdmCl solution (pH 2.3). Added 10 eq. MPAA and 5 eq. Pentanedione. Adjusted the pH to 2–2.5. Allow the reaction to proceed at room temperature for 2 h.

#### Native chemical ligation (NCL)^52^

Dissolved the hydrazide peptide fragment (1 mM) in 6 M GdmCl solution (pH 2.3). In an ice salt bath at –20 °C, added 10 eq. NaNO_2_ (0.5 M). Allowed oxidation to proceed for 20 min. Then, added 50 eq. MPAA and 1.1 eq. the N-terminal Cys peptide fragment. Adjusted the pH to 6.3–6.5 using a glass electrode. Allowed the reaction to proceed at room temperature overnight.

#### Desulfurization^53^

Dissolved the peptide fragment (1 mM) in 6 M GdmCl and 0.5 M TCEP solution (pH 7). Added 100 eq. tBuSH and 100 eq. VA044. Adjusted the pH to 7–7.2. Allowed the reaction to proceed at room temperature for 3 h.

#### One-pot ligation and desulfurization

Dissolved the MPAA thioester peptide fragment (1 mM) and the N-terminal Cys peptide fragment (1.1 mM) in 6 M GdmCl (pH 7). Adjusted the pH to 6.3–6.5. Allowed the reaction to proceed at room temperature overnight. Then diluted the mixture threefold with a 300 mM TCEP solution (pH 7). Added 10% (v/v) tBuSH and 40 eq. VA044. Adjusted the pH to 7–7.2, and allowed the reaction to proceed at room temperature for 3 h.

#### Remove Acm^54^

Dissolved the peptide fragment (1 mM) in 6 M GdmCl (pH 7). Added 15 eq. PdCl_2_ and TCEP (5 mg/mL). Adjusted the pH to 7–7.2, and allowed the reaction to proceed at room temperature for 2 h. Finally, added DTT for quenching.

#### Remove Allyl^55^

Dissolved the peptide fragment (1 mM) in NCL solution (6 M GdmCl, 100 mM MPAA, 40 mM TCEP, pH 7). Added 3 eq. [Pd(Allyl)Cl]_2_, adjusted the pH to 7–7.2. Allowed the reaction to proceed at room temperature for 2 h. Finally, added DTT for quenching.

#### Removal of RBM^38^

The RBM group on peptide was cleaved with TFA cocktails (TFA/TIPS/water, 95:2.5:2.5, v/v/v) for 3 h. The TFA solution was then concentrated under nitrogen agitation, followed by the addition of acetonitrile in water. The solution was subsequently lyophilized.

#### Synthetic route of d-19437_l-Pep-1_

d-19437_l-Pep-1_ was obtained by standard Fmoc SPPS. The peptide was eluted from a C18 column (4.6 × 250 mm) using a linear gradient of 30%–70% Buffer A in B for over 30 min. RP-HPLC isolation yield was 5% (Supplementary information, Fig. S9).

#### Synthetic route of d-25367_l-IL-6_

d-25367_l-IL-6_ was obtained by standard Fmoc SPPS. The peptide was eluted from a C18 column (4.6 × 250 mm) using a linear gradient of 30%–80% Buffer A in B for over 30 min. RP-HPLC isolation yield was 4.5% (Supplementary information, Fig. S9).

#### Synthetic route of d-57445_l-TrkA_

d-57445_l-TrkA_ was obtained by standard Fmoc SPPS. The peptide was eluted from a C18 column (4.6 × 250 mm) using a linear gradient of 30%–80% Buffer A in B for over 30 min. RP-HPLC isolation yield was 6.7% (Supplementary information, Fig. S9).

### Protein purification

#### Expression, purification and refolding of TrkA

The D5 domain of human TrkA (l-TrkA, residues 283–384) was cloned into the pET-21a vector with an N-terminal 6× His tag. The construct was transformed into competent Lemo21(DE3) cells. For l-TrkA, the *E. coli* cells were grown in LB medium at 37 °C until the OD_600_ reached 0.6–0.8. IPTG was added to a final concentration of 1 mM and the cells were incubated for 3–4 h at 37 °C. Collected cells were resuspended in 10% glycerol and sonicated, and the inclusion body was purified and successively washed by buffer A (1% TritonX-100, 10 mM Tris-HCl, pH 8.0, 1 mM EDTA), buffer B (1 M NaCl, 10 mM Tris-HCl, pH 8.0, 1 mM EDTA) and buffer C (10 mM Tris-HCl, pH 8.0, 1 mM EDTA). The purified inclusion body was dissolved in buffer D (8 M Urea, phosphate-buffered saline (PBS), pH 7.4, 30 mM imidazole). The supernatant was collected from centrifugation (13,000× *g* for 1 h), and applied to Nickel Sepharose 6 Fast Flow resin for affinity purification. The eluate was diluted in buffer D with 2.5 mM DTT to a final protein concentration of 0.1 mg/mL. After dialysis in buffer E (20 mM Tris-HCl, pH 8.5, 50 mM NaCl) for 24 h, the protein was concentrated and further purified by SEC (Superdex 75 Increase). All protein samples were characterized by SDS-PAGE, with purity greater than 95%. d-TrkA was refolded using the same protocol.

#### Expression, purification and refolding of IL-6

The gene encoding human IL-6 (28-212, with an additional methionine at the N-terminus) was cloned into the pET-28b vector, and the resulting plasmid was then transformed into competent Lemo21(DE3) cells. *E. coli* cells were grown in LB medium at 37 °C until the OD_600_ reached 1.0. IPTG was added to a final concentration of 1 mM and the cells were incubated at 37 °C for 3–4 h. Cells were harvested by centrifugation at 10,000× *g*. The pellet was resuspended in PBS (pH 7.4), and disrupted by sonication. The lysates were centrifuged at 20,000× *g* for 1 h at 4 °C. The pellets containing the inclusion body of IL-6 were resuspended in a buffer containing 6 M GdmCl, 0.1 M Tris-HCl, pH 8.0, and 30 mM imidazole, and then centrifuged at 20,000× *g* for 1 h at 4 °C. The supernatant was applied to nickel resin (Ni Sepharose 6 Fast Flow, Absin) for affinity purification. Purified protein in a buffer containing 6 M GdmCl, 0.1 M Tris-HCl, pH 8.0, and 300 mM imidazole was incubated with 10 mM dithiothreitol (final concentration) at room temperature for 1 h, and was diluted into PBS (pH 7.4) containing 2 mM reduced glutathione and 0.2 mM oxidized glutathione to a final protein concentration of 0.25 mg/mL. The mixture was incubated at room temperature for 2 h followed by a dialysis against PBS buffer (pH 7.4) at 4 °C for 24 h. The dialyzed protein was concentrated and purified by SEC (Superdex 200 increase column). d-IL-6 was refolded using the same protocol.

#### Expression and purification of l-protein binders

The genes encoding binders l-19437_d-Pep-1_, l-57445_d-TrkA_ and l-25367_d-IL-6_ were amplified from the corresponding yeast library, cloned into the pET-28b vector containing an N-terminal 6× His tag and a TEV protease cutting site, and sequenced. The constructs were transformed into competent Lemo21(DE3) cells. Cells were grown in LB medium at 37 °C until OD_600_ reached 0.6, and protein expression was induced at 18 °C overnight by addition of 0.1 mM IPTG (final concentration). Harvested cells in PBS buffer containing 500 mM NaCl (pH 7.4) were lysed by sonication and centrifuged. The protein in supernatant was extracted using a gravity flow column containing 1 mL Ni Sepharose 6 Fast Flow resin (Cytiva Life Sciences), purified by SEC (ÄKTA pure, Cytiva Life Sciences) on a Superdex 75 Increase 10/300 GL column (Cytiva Life Sciences) in PBS buffer (pH 7.4), and verified by SDS-PAGE. Peak fractions were pooled, concentrated, snap-frozen by liquid nitrogen and stored at –80 °C.

#### Folding of d-protein binders

Chemically synthesized d-19437_l-Pep-1_, d-57445_l-TrkA_, and d-25367_l-IL-6_ were dissolved in denaturing buffer containing 6 M GdmCl and 100 mM Tris-HCl (pH 8.0) to 0.1 mg/mL and dialyzed against TBS buffer (pH 7.4) for 6 h. The proteins were dialyzed against fresh TBS buffer for another 12 h. Dialyzed sample was concentrated and purified by SEC on a Superdex 75 Increase 10/300 GL column (Cytiva Life Sciences) in PBS buffer (pH 7.4) and verified by SDS-PAGE. Peak fractions were pooled, concentrated, snap-frozen by liquid nitrogen and stored at –80 °C.

### Biochemical and biophysical characterization

#### CD

CD data were collected in a 0.5-mm path-length cuvette from a Chirascan V100 CD spectrometer (AppliedPhotophysics). CD spectra of targets were measured from 260 nm to 180 nm in triplets and averaged. For designer binders, CD spectra were applied at various temperatures from 25 °C to 95 °C. Temperature melts were conducted in 2 °C steps (heating rate of 2 °C/min) by measuring the signal at a wavelength of 222 nm. The concentrations of proteins measured in CD were in the range of 0.1–0.4 mg/mL.

#### Biolayer interferometry

Biolayer interferometry binding data were collected and processed using Octet RED96e (ForteBio). All target proteins (5–10 μg/mL) were biotinylated and immobilized onto streptavidin-coated biosensors (SA Sartorius) in a binding buffer containing PBS (pH 7.4) with 0.02% TWEEN. The biosensors were equilibrated in binding buffer for 60 s and then dipped into binder solution for 180 s or 300 s (association step), and then dipped into binding buffer (dissociation step). The experiment was carried out at 25 °C. In the cross-reactivity assay, binding of 1 μM different miniprotein binders to l-Pep1, l-TrkA and l-IL-6 were tested, respectively. All the experiments were repeated twice with similar results. The data were analyzed using the global fitting algorithm provided in the Octet data analysis software, DataAnalysisHT.

#### ITC

Binding affinity determination by ITC was performed on a MicroCal PEAQ-ITC (Malvern) with a 19-drop method following instruction of the instrument. The first drop was 0.4 μL and each drop of the rest was 2 μL. The spacing between drops was 150 s. The analysis of results was performed using the MicroCal PEAQ-ITC Analysis Software. In this process, the evaluation of l-19437/d-Pep-1 and d-19437/l-Pep-1 was conducted using the One Set of Sites mode, while the assessment of d-25367-evo/l-IL-6 was carried out employing the Two Sets of Sites mode. The first drop was excluded during analysis as default. In the ITC titration, l-19437 (15 μM) and d-Pep-1 (150 μM), along with d-25367-evo (575 μM) and l-IL-6 (57.5 μM), were utilized in a PBS solution at a pH of 7.4.

#### MST

MST experiments were conducted using a Nanotemper Monolith equipped with the NT.115 Capillaries. The MST curves were acquired under identical conditions, with a protein concentration of 50 nM l-TrkA in a buffer composed of PBS with 0.05% Tween20 at pH 7.4. A total of 16 different ligand concentrations were utilized for each measurement, ranging from 122 pM to 40 μM for d-57445-evo_l-TrkA_. These concentrations were achieved through serial dilution with a factor of 2. The laser power of the MST experiment was set to 40%, and the acquired data were analyzed using MO.Control v1.6.1 acquisition software.

#### Co-migration on SEC

The interaction between the targets (l-Pep-1, l-TrkA and l-IL-6) and the cognate binders (d-19437, d-57445, and d-25367), were examined by SEC analysis. Protein solution for targets, binders and target–binder mixtures was applied to SEC, respectively. l-Pep-1, d-19437, l-Pep-1 + d-19437, l-TrkA, d-57445 and l-TrkA + d-57445 were separated on a Superdex 75 Increase 10/300 column. l-IL-6, d-25367 and l-IL-6 + d-25367 were subjected into a Superdex 200 Increase 10/300 column. Co-migration of proteins was examined by SDS-PAGE.

#### Protease stability assay

In the digestion system, the final concentrations of l- and d-proteins were adjusted to 0.2 mg/mL. The final concentration of trypsin (Genom) was 2.2 mg/mL in the Hank’s Balanced Salt Solution with 0.02% EDTA. The final concentration of Pepsin (Aladin) was 0.22 mg/mL in a buffer containing 0.1 M Glycine, pH 2.5. Digestion was performed at 37 °C.

### Yeast display

#### Library preparation

All designer sequences were extended to 65 amino acids using a (GS)n linker after the coding sequences of the designs for better polymerase chain reaction (PCR) amplification. The protein sequences were reversed translated and codon optimized using DNAworks 2.0^56^ for expression in *Saccharomyces cerevisiae*.^7^

#### Gene pools

Oligonucleotide pools (Agilent) were constructed following a published protocol.^9^ Briefly, genes were flanked with a common 18-bp adaptor at 5′ and with a 17-bp adaptor at 3′ for amplification. The oligonucleotide pools were amplified with 2× PCR Master Mix (KOD One) using extension primers to add one pETCON vector homologous recombination segment (40 bp) to each end. Amplified pools were loaded on a 1.5% agarose gel and the bands with expected size were extracted (ComWin Biotech Gel Extraction Kit). pETCON backbone was linearized by PCR and gel extracted, producing 3–4 μg DNA. *S. cerevisiae* EBY100 cells were transformed with the library DNA and linearized pETCON vector using an established protocol,^57^ but the amount of library DNA was adjusted to 4 μg instead of 16 μg. After transformation (at least 1 × 10^7^ transformants), yeast cells were grown overnight in 60 mL SDCAA medium at 30 °C and were concentrated to OD_600_ 10 followed by mixing with an equal volume of 50% (v/v) glycerol and stored in 2 mL aliquots at –80 °C.

#### Yeast display and deep sequencing

Yeast display was based on an established protocol.^58^ 1 × 10^7^ yeast cells were inoculated into 10 mL SDCAA medium and were grown at 30 °C until OD_600_ reached 2–5. Cells were then centrifuged at 3000× *g* for 3 min, resuspended in 10 mL SGCAA, and induced at 20 °C for 20–28 h. Then, yeast cells were concentrated to 10^8^ cells/mL and incubated with 1:250 diluted anti-c-Myc fluorescein isothiocyanate (FITC, Miltenyi Biotech) and biotinylated targets of various concentrations. Next, the cells were incubated with 1:100 diluted streptavidin-phycoerythrin (SAPE, R&D Systems). The cells were washed with 0.5 mL PBSF (PBS with 0.1% (w/v) bovine serum albumin (BSA)) right before and after each incubation step. The labeled cells were sorted on a sorter (BD Melody). The concentrations of targets were: 100 nM and 10 nM for 2 rounds of sorting of d-Pep-1 binders; 5 μM and 500 nM for 2 rounds of sorting of d-TrkA binders; 1 μM, 100 nM, 10 nM and 1 nM for 4 rounds of sorting of d-IL-6 binders. 2 × 10^3^–5 × 10^4^ cells with strongest double fluorescence signal were collected after each round and were grown in 5 mL SDCAA medium at 30 °C until OD_600_ reached 2–5; at least 1 × 10^8^ cells were spun down at 13,000× *g* for 1 min and stored as cell pellets at –80 °C for plasmid extraction. Yeast plasmids were extracted (TIANprep Yeast Plasmid DNA Kit) and eluted in 70 μL distilled water, serving as templates for deep sequencing. Deep sequencing libraries with library-specific barcodes were prepared by PCR amplification for 22–30 cycles in a 50 μL KOD One reaction system. The PCR products were separated on a 1.5% agarose gel and extracted (ComWin Biotech Gel Extraction Kit). Purified DNA was mixed and sent for deep sequencing (Novogene). For the d-Pep-1 pool, after sorting against d-Pep-1 at concentrations of 100 nM and 10 nM, we identified 140 and 11 diverse sequences, respectively. For the d-TrkA pool, sorting was performed at d-TrkA concentrations of 5000 nM and 500 nM, which yielded 85 and 37 distinct sequences, respectively. After sorting at d-IL-6 concentrations of 1000 nM, 100 nM, 10 nM and 1 nM, the enriched populations contained 431, 143, 65 and 61 different sequences, respectively.

#### Entropy score

The entropy score for each position of the binder was calculated as follows:

$${H}_{i}\,=\,-{\sum}_{j\,=\,1}^{20}P\left({x}_{i,j}\right){\log }_{2}P\left({x}_{i,j}\right),\,i\,=\,1,2,\ldots ,\,K$$

${x}_{i,j}$ represents a mutant protein binder with the i^th^ residue mutated to j (one of the 20 amino acids). $P\left({x}_{i,j}\right)$ is the observed frequency of ${x}_{i,j}$ from the NGS data. ${{{\rm{K}}}}$ is the length of the protein binder. For the d-TrkA binder l-57445_d-TrkA_, NGS data from the 1^st^ round of selection in the site saturation mutagenesis experiment were used to calculate the entropy score. Positions with lower entropy score are more conserved, and vice versa.

#### Combo library

To introduce combinatorial mutations, degenerate codons were utilized to designate different amino acids for each beneficial position, based on the results of the SSM analysis. The DNA fragments necessary for this process were generated through PCR, employing library primers that carried the degenerate codons. The ratio used for mixing the primers ensured that each encoding amino acid appeared with equal frequency. Subsequently, DNA insert fragments were yielded through PCR amplification, utilizing primers that contained overlap with the previously linearized pETCON vector. Following this, the DNA insert fragments were purified through gel electrophoresis and combined with the linearized pETCON vector in preparation for transformation.

#### Error-prone PCR library construction

In order to create the binder library, error-prone PCR was performed using the GeneMorph II Random Mutagenesis Kits (Agilent). The PCR reaction included a mutation rate of 1–3 amino acids per gene. Once the libraries were successfully reconstructed, they were transferred into *S. cerevisiae* EBY100 cells.

### Cell-based assay

#### TF-1 cell proliferation assay^7^

TF-1 cells were incubated with different concentrations of d-57445-evo_l-TrkA_ and NGF in the RPMI-1640 media containing 2% FBS for 48 h at 37 °C with 5% CO_2_. Cell proliferation was assessed by measuring cellular ATP level using ApoSENSOR™ Cell Viability Assay Kit (BioVision) according to the manufacturer’s protocol. Luminescent signal was measured by using a Thermo Varioskan LUX microplate reader, and the data were plotted and analyzed by Prism 8 (GraphPad).

#### Detection of the phosphorylation of Akt and Erk kinases

After a 4-h period of starvation treatment, TF-1 cells were incubated with NGF at a concentration of 100 ng/mL and treated with various binders. This incubation process took place at a temperature of 37 °C for a duration of 10 min. Subsequently, the cells were collected through centrifugation and subjected to cell lysis. The lysate of the whole cell protein was obtained and analyzed via western blot analysis to assess the protein phosphorylation levels. This analysis was conducted using the PhosphoPlus Akt (Ser473) Antibody Duet kit and the PhosphoPlus p44/42 MAPK (Erk1/2) (Thr202/Tyr204) Antibody Duet kit, both from Cell Signaling Technology.

#### IL-6 signaling assay^59^

The production of human placental secreted alkaline phosphatase (SEAP) in cell culture medium was described previously.^59^ Briefly, 1 × 10^4^ HEK-293T cells were transfected with P_hCMV_-hIL-6R-pA (40 ng), P_hCMV_-hSTAT3-pA (40 ng) and P_hSTAT3_-SEAP-pA (20 ng) to produce SEAP in response to human IL-6. The transfected cells were incubated with the different concentrations of d-25367-evo_l-IL-6_ and human IL-6 in DMEM medium containing 10% FBS for 48 h at 37 °C with 5% CO_2_. IL-6 signaling was assessed by measuring the production of SEAP in the cell culture medium. The cell culture supernatant was heat-inactivated (65 °C for 30 min), and 80 µL supernatant was mixed with 120 µL of substrate solution (100 µL of 2× SEAP assay buffer containing 20 mM homoarginine, 1 mM MgCl_2_, 21% (v/v) diethanolamine (pH 9.8), and 20 µL of substrate solution containing 120 mM p-nitrophenylphosphate). Absorbance was recorded at 405 nm (37 °C) using a Synergy H1 hybrid multimode microplate reader (BioTek Instruments Inc.). The data were plotted and analyzed by Prism 8 (GraphPad).

### Crystallography

#### Crystallization

Purified l-19437 (8 mg) was incubated with TEV protease (2 mg) at room temperature for 4 h to remove His tag. The cleaved product was purified by using a gravity flow column containing 1.5 mL Nickel Sepharose 6 Fast Flow resin (Cytiva Life Sciences) in TBS buffer (pH 7.4). 3.5 mg l-19437 was incubated with 1.25 mg d-Pep-1 and purified by SEC on a Superdex 75 Increase 10/300 column (Cytiva Life Sciences). The complex was concentrated to 8.1 mg/mL and subjected to crystal screen (hanging-drop method, with a protein/buffer ratio of 1:1) by using a Mosquito crystallization robot (SPT Labtech). Prism-like crystals grew into full size after 45 days. The crystallization condition contained 20% (w/v) PEG 8000, 100 mM Tris-HCl (pH 8.5), 100 mM magnesium chloride and 20% (v/v) PEG 400. Crystals of d-19437 and l-Pep-1 were grown in the same crystallization condition.

#### Data collection and structure determination

Crystals were cryoprotected by addition of 15% glycerol. Diffraction data at 2.2 Å resolution were collected at 100 K by using an in-house X-ray generator (XtaLAB Synergy Custom diffractometer, Rigaku). Crystals were in space group of P2_1_2_1_2. Data were indexed, integrated and scaled using HKL-2000.^60^ Further processing was carried out with programs from the CCP4 suites.^61^ Data collection statistics are summarized in Supplementary information, Table S1. MR solution was found by Phaser^62^ using the design model as the searching model. Two copies of the designed heterochiral complexes in one asymmetric unit (with translational non-crystallographic symmetry) were identified.

## Supplementary information

Supplementary information, Fig. S1

Supplementary information, Fig. S2

Supplementary information, Fig. S3

Supplementary information, Fig. S4

Supplementary information, Fig. S5

Supplementary information, Fig. S6

Supplementary information, Fig. S7

Supplementary information, Fig. S8

Supplementary information, Fig. S9

Supplementary information, Fig. S10

Supplementary information, Fig. S11

Supplementary information, Fig. S12

Supplementary information, Fig. S13

Supplementary information, Fig. S14

Supplementary information, Fig. S15

Supplementary information, Table S1

Supplementary information, Table S2

Supplementary information, Table S3

Supplementary information, Table S4

Supplementary information, Table S5

Supplementary information, Dataset S1

## Data Availability

The crystal structure models have been deposited in the PDB under accession codes: 8GQP for d-19437–l-Pep-1; 7YH8 for l-19437–d-Pep-1. All data are available in the main text or the supplementary materials. Design scripts are available in Supplementary information, Dataset S1.
