# Supplementary material for: Accurate de novo design of heterochiral protein–protein interactions
Source: Cell Res. 2024 Aug 14;34(12):846–58. doi: 10.1038/s41422-024-01014-2 (PMC11614891; doi:10.1038/s41422-024-01014-2)
Supplement: Supplementary file 1 — Supplementary information, Fig. S1 [file 41422_2024_1014_MOESM1_ESM.pdf]

Supplementary Figures

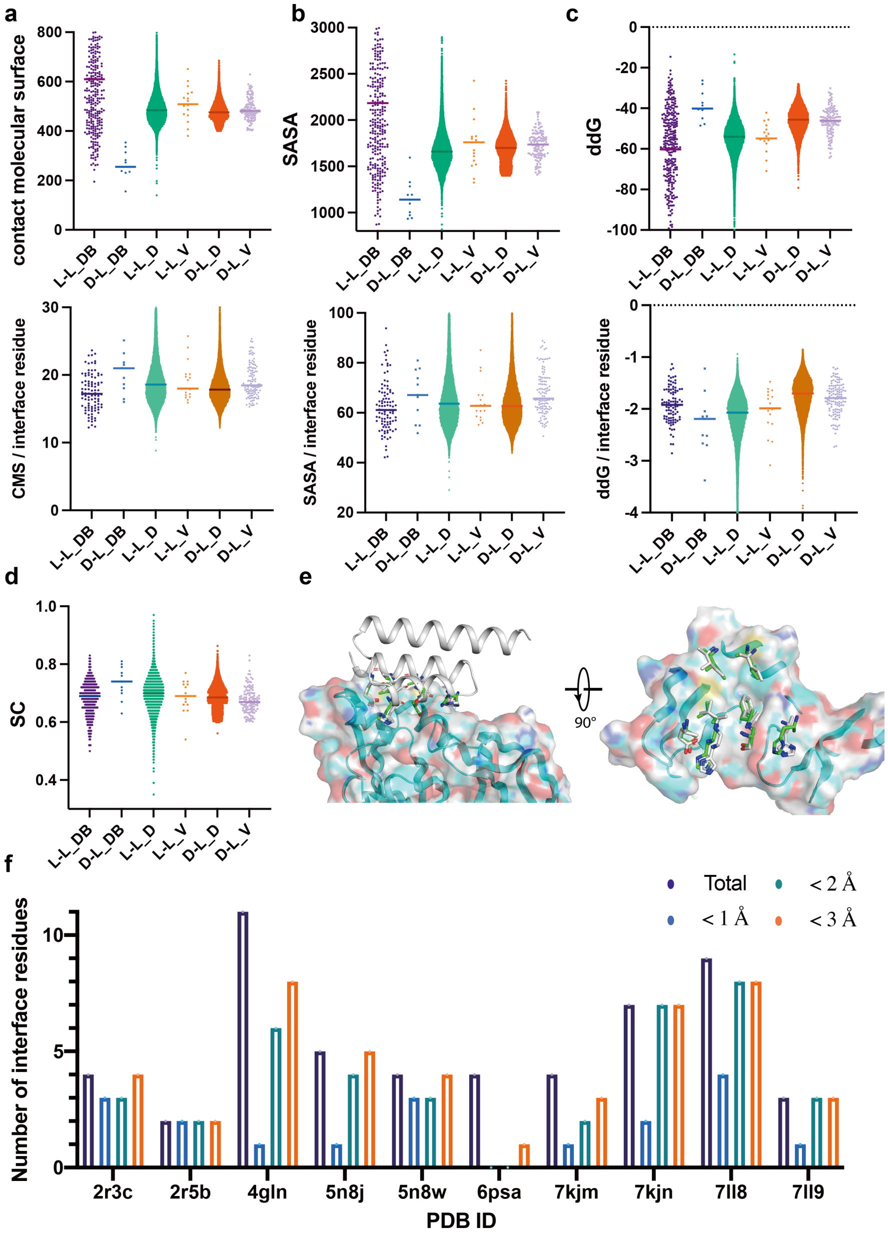

Fig. S1 | Comparison of the computational interface metrics for hetero-chiral and homo-chiral protein complexes.

Interface metrics, such as the contact molecular surface (CMS, (**a**)), the solvent-accessible surface area (SASA, (**b**)), the Rosetta binding energy (dd$G$, (**c**)), and the shape complementarity (SC, (**d**)), were calculated for both hetero- and homo-chiral protein complexes. L-L, homo-chiral; D-L, hetero-chiral. DB, structures of protein complexes available in the protein data bank. We

calculated the interface metrics for 95 and 10 structure models of L-L_DB[62] and D-L_DB, respectively. We collected the heterochiral protein complexes with unique sequences in the PDB. D, *de novo* designed protein complexes. V, *de novo* designer protein complexes verified in experiments. Interface metrics for ~30,000 and 17 structure models (validated for *in vitro* target binding) of L-L_D and L-L_V were calculated, respectively. We calculated the interface metrics for ~38,000 and 120 structure models (designs enriched from the yeast display experiments) of D-L_D and D-L_V, respectively. Individual values were shown in the scatter dot plots with bars representing median values. We used the Rosetta energy function "beta"[41,42] to calculate the energetics. **e**, RifDock accurately recovered a substantial number of D-protein target interacting L-amino acids from the D-protein target structure. RFX-V1 is a D-protein selected by using mirror-image phage display against human vascular endothelial growth factor A (VEGF-A)[14]. D-VEGF-A is shown in surface and cartoon representation in cyan, and L-RFX-V1 is shown in gray. Interface residues of L-RFX-V1 are highlighted in sticks. L-amino acids interacting with D-VEGF-A recovered by RifDock (green) with the lowest RMSD values from the native interacting residues (gray) are shown. **f**, Recovery of the interacting L-rotamers from D-protein target structures by RifDock. Total numbers of interface residues from the binders are shown (total). We docked discrete L-amino acids against interface regions of D-protein target by using RifDock and tallied recovered residues with all atom RMSD values in different ranges.
