# Supplementary material for: Accurate de novo design of heterochiral protein–protein interactions
Source: Cell Res. 2024 Aug 14;34(12):846–58. doi: 10.1038/s41422-024-01014-2 (PMC11614891; doi:10.1038/s41422-024-01014-2)
Supplement: Supplementary file 2 — Supplementary information, Fig. S2 [file 41422_2024_1014_MOESM2_ESM.pdf]

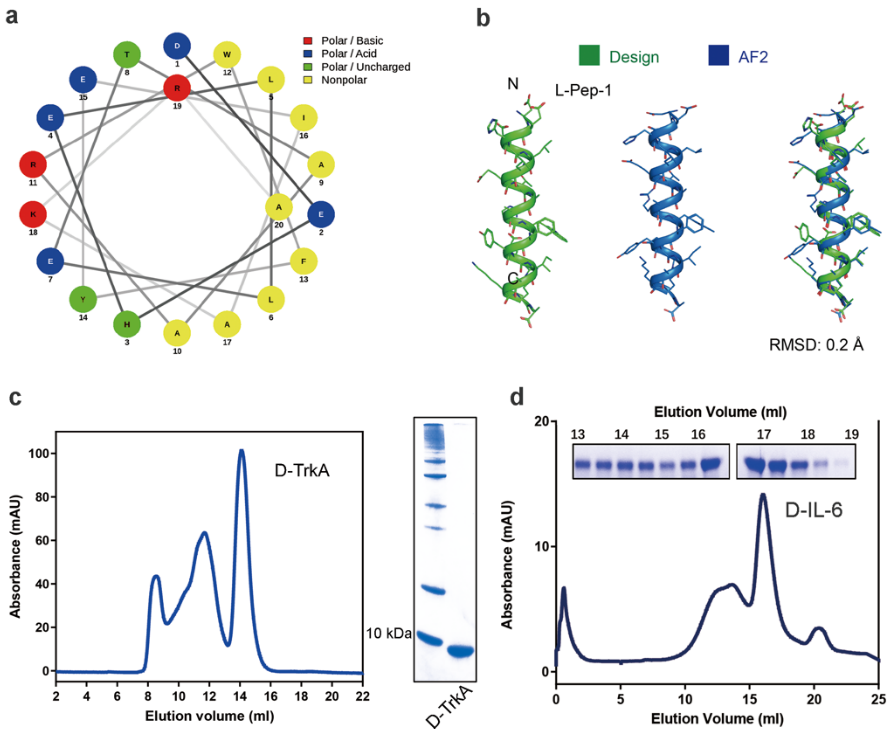

Fig. S2 | Preparation of targets.

(**a** and **b**) Preparation of L-Pep-1. **a**, Helical wheel[61] representing L-Pep-1. Hydrophobic residues are in yellow. **b**, Prediction of the 3D structure of L-Pep-1 by using AlphaFold2. Green, design model. Blue, AF2 model. The design model and the AF2 model are almost identical, with a Cα RMSD of 0.2 Å. **c**, Representative gel filtration chromatography and SDS-PAGE of folded D-TrkA. **d**, Representative gel filtration chromatography and SDS-PAGE results of folded D-IL-6.
