# Supplementary material for: Accurate de novo design of heterochiral protein–protein interactions
Source: Cell Res. 2024 Aug 14;34(12):846–58. doi: 10.1038/s41422-024-01014-2 (PMC11614891; doi:10.1038/s41422-024-01014-2)
Supplement: Supplementary file 3 — Supplementary information, Fig. S3 [file 41422_2024_1014_MOESM3_ESM.pdf]

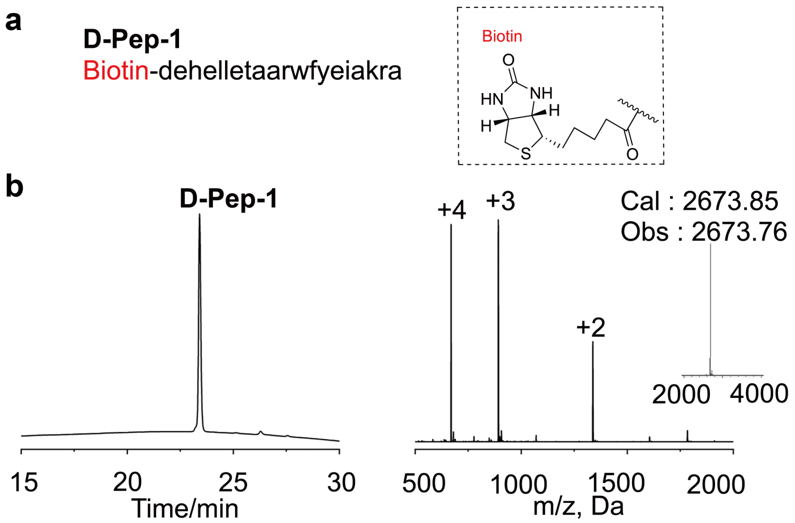

Fig. S3 | Synthesis of D-Pep-1.

a, Amino acid sequence of D-Pep-1; **b,** HPLC analysis and ESI-MS results of purified D-Pep-1.
