# Supplementary material for: Accurate de novo design of heterochiral protein–protein interactions
Source: Cell Res. 2024 Aug 14;34(12):846–58. doi: 10.1038/s41422-024-01014-2 (PMC11614891; doi:10.1038/s41422-024-01014-2)
Supplement: Supplementary file 4 — Supplementary information, Fig. S4 [file 41422_2024_1014_MOESM4_ESM.pdf]

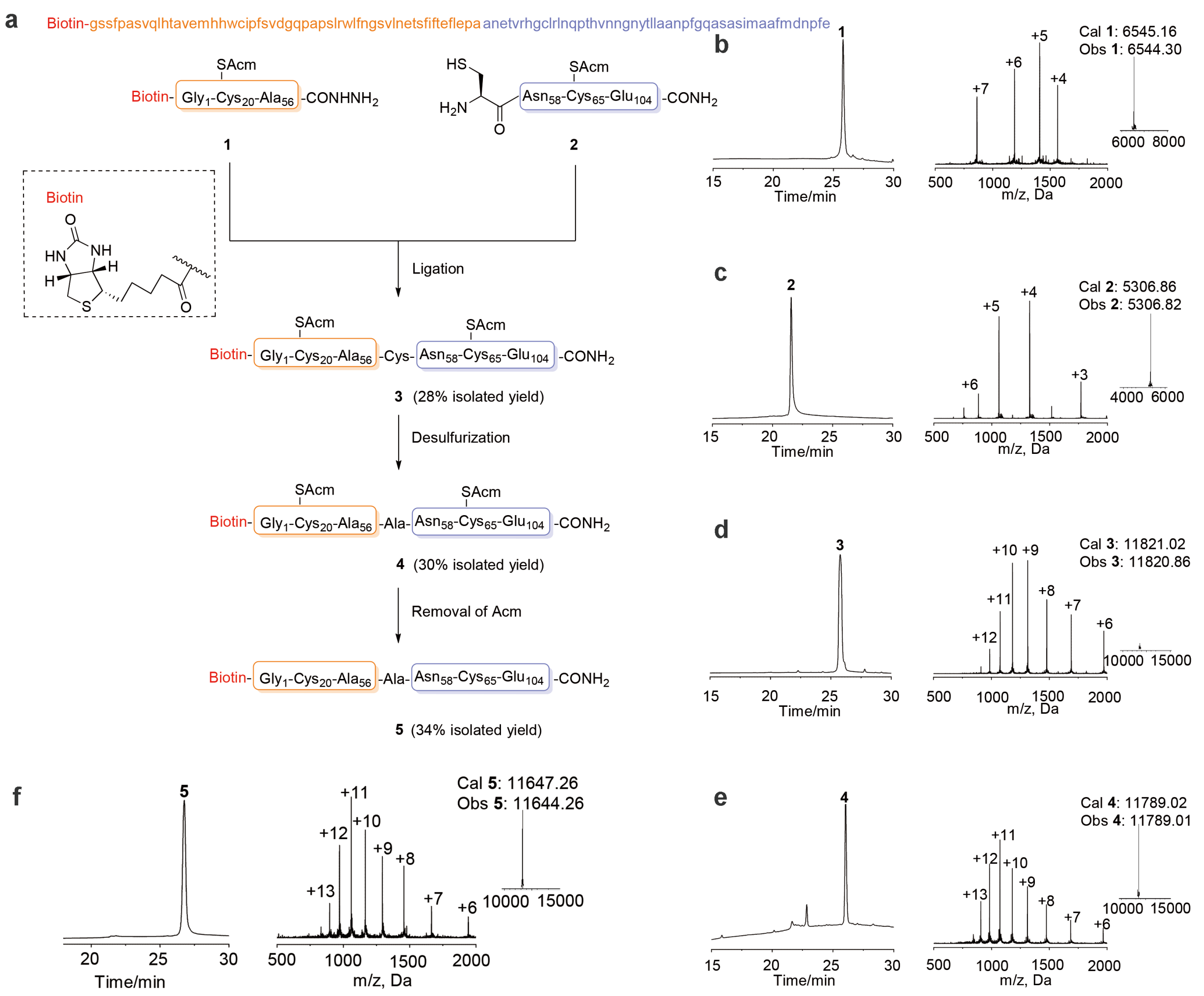

Fig. S4 | Synthetic route of D-TrkA.

a, Chemical total synthesis route of D-TrkA; (**b-f**). HPLC analysis and ESI-MS results of purified **1-5**.
