# Supplementary material for: Accurate de novo design of heterochiral protein–protein interactions
Source: Cell Res. 2024 Aug 14;34(12):846–58. doi: 10.1038/s41422-024-01014-2 (PMC11614891; doi:10.1038/s41422-024-01014-2)
Supplement: Supplementary file 5 — Supplementary information, Fig. S5 [file 41422_2024_1014_MOESM5_ESM.pdf]

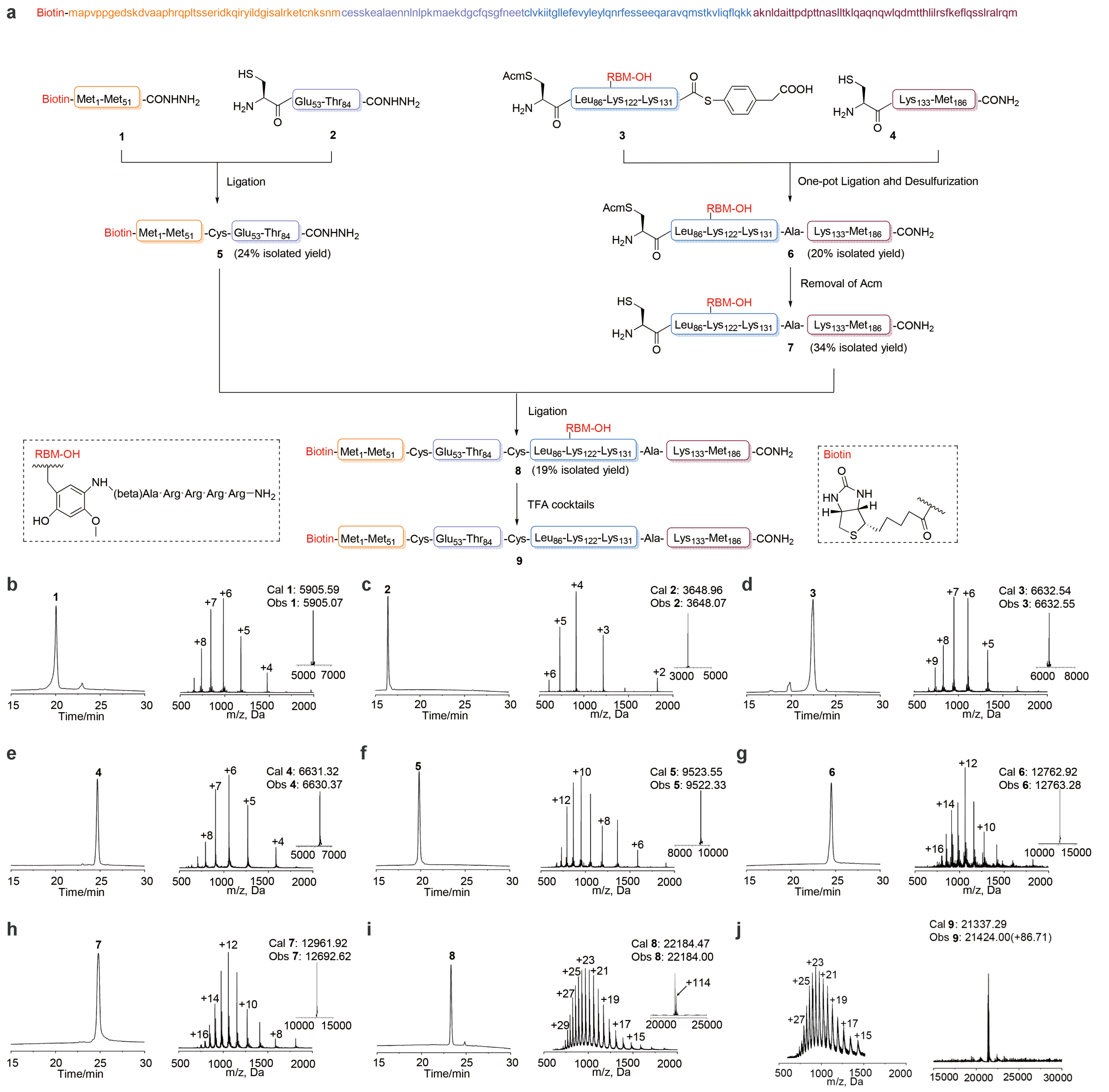

1

2 **Fig. S5 | Synthetic route of D-IL-6.**

3 **a,** Chemical total synthesis route of D-IL-6; **b-j,** HPLC analysis and ESI-MS results of purified **1-**

4 **9**.

5
