# Supplementary material for: Accurate de novo design of heterochiral protein–protein interactions
Source: Cell Res. 2024 Aug 14;34(12):846–58. doi: 10.1038/s41422-024-01014-2 (PMC11614891; doi:10.1038/s41422-024-01014-2)
Supplement: Supplementary file 6 — Supplementary information, Fig. S6 [file 41422_2024_1014_MOESM6_ESM.pdf]

a Pep1

```
                        10        20        30        40        50        60        70        80
19437(HHH)      ----------LPVEKIIREAKKILDELLKRG--------LIDPELARIAREVLERARKLG-NEEAARFVLELIERLRRELS-
22837(HHH)      ----------LPVEKIIREAKKILDELLKRG--------LIDPELARIAREVLERARKLG-SEEAARFVLELIERLRRRLS-
22859(HHH)      ----------SEVEEASRRIKKLLDEALKRG--------LISPRAVRFVKELVEEARKLG-NEKALKFALEWIREFIERQ--
22842(HHH)      ----------LEIEKELKKLLKEVEKLVRKG--------LVSPEFVEIVREVIERAKRLG-SERALEFVRRLIERAIREIE-
19120(HHHH)     SVEELTELIKKALEEGDPELLKRLIELLKEL--------IGDPKLRKYAEEILERAIRLG-SPEVLEFIVRLVS--------
21276(HHH)      ------SEIKKKWEEVSKKLQKIADQLSPRQR-------KAVKRLIEIARRLLERAR----SEETKEFVLRLLERAVKLII-
17960(HHH)      -----------DQEEVEKKLETIRKEAEKRND-------ERKYELADFLLRLFRRALEQVRNEEARKIIIEIAKRLAERAS-
22539(HHH)      ---------GEAEQEVARIAAEALTEAIKRN----------NERLLRFVIKLAKRALKLIKDPTARKILKEAIEKAEKVVKG
20240(HHH)      ------------SEETAKRLLKIVEKLAKKVGT------RAAEFVLELARRLLKELKEGKLSEEAVERALKIAIELLRRAG-
16544(HHHH)     -----------SWREVAERLQRLNTPVARIAAALLFALLLGDEELVERFRRRLEELSKEG--DPEAREVLELVRRLIP----
22527(HHH)      ----------SQKDEAQKLVKKAIEIAQRRN-------LEEVARLLEALLRAVREFARYAPNERIVRWAAEHLASHILSL--
```

b TrkA

```
                        10        20        30        40        50        60        70        80
55816(HHHH)     --DEILEEIEEALRRGDPELV--------------ERLIEKLEKLAKEL--GDPKLERAARILRRLLKYNPLFVFLYLRQVRK-
55594(HHHH)     TRREIFRLLREAREEGDEELLRVLRQAYIAFLTDEDRAIEFAEKLAKEL--GDPRLEELVELLRKLK-----------------
55826(HHHH)     --PEIERAEELARKLGDPFLFLYLRQLRELLEEGDEERVERARDAASQL--AKRVNSPLAREILELIEK---------------
55883(HHHH)     -PPELIEKLEELLRKGD---------------PRAEEVVRRLYELAERL--GDPHLSRLAIFLYLYIRQGSFRAALFLLEQLP-
55810(HHHH)     --DREEEHLERLKKFLSPELV---------------ERLKEILKRLREK--GDPRRVFVLLYIRQANKGSPRALRVLEQIARRT
55851(HHHH)     --DKEEERIRRLLKRLG----------------VSPEVIERVIELIKRIKKGDPEAKRLVEELIRELAKRNLFVALYVRQLIK-
55954(HHHH)     --SEEYEELLELISRVSPFLR---------------RLAKLLEEALRRN--DKELVHRLLVFIALYLRQRGDPVFEVVRRLLEK
55748(HHHH)     SFRELVQLVLRLLMRKNPQLA---------------FYILQLLDRAIRR--GDPKLVIEIAVKAAELVGDPELLEKLRRLLK--
59560(HHHH)     DLKRLLAKVLARLFRLDP------------------RLFRELMKVDRIFQ-KDPETALRIVLELVRRLGDEELEELVKRLLEKT
58678(HHHH)     ---SLLERVVELLRKVDPEVA---------------RYVEEFIKRLREQGIDPRIIAVLVAVQLRRRGLLTPEIRELLQRLAK-
57445(HHHH)     STEELVEELLELARKDPQEAR---------------KLVEEIVKELGLS----PERRFELQLAIVLAQRDPELARRILERALNH
```

c IL6

```
                        10        20        30        40        50        60        70
24534m(HHHH)    ---SEELLEIIKELLKRGISPE-EVVEILVKIVEELGDP--DLLTFVQLFESLVHIIGDPEKAVKLLEEET-
24534(HHHH)     ---SEELLEIIKELLKRGISPE-EVVEILVKIVEELGDP--DLLTFVQLFESLVHITGDPEKAVKLLEEET-
25367(HHHH)     ------GEEEVKEYLTRKFKDDPELVRLLREAIEVLLKAGEDPELVLSLIESLIHITGDPRAAVKLAKEFG-
34837(HHHH)     ---SEKEEEVIRILSELRGVSPEEIRRLLEELERELGDP---ETALYQLVVWVYIVTGDPEVHKLLEKVDK-
34305(HHHH)     ---SEKEEEVIRILSELLGVSPEEIRRLLEELERELGDP---EQALYQLVVWVYIVLGDPEVHKLLEKVDK-
38955(HHHH)     ---DEKEEVRKRFEELVRQGNPEEALFLVLIFLAELNTP----EARELYVKLFALRLRDPEKALELAKEVLR
34548(HHHH)     ------SEELSEKIEEALKRGDEELVYQLVVWLYLLAYYRGDEELLEELEKVTELLHRDPEKAVRLVLRLT-
30405(HHHH)     ----DELDELVKLIVELVGLSPEEQKLLEELAEEAKREG--NPDLALSFLWLIAYYRGDEELLRVLEKLYK-
31812(HHH)      YYLQESARFVARELTKAIRDNDEELVRLLEEQLEILLALAEEENNEEVVKYIRQLIEEVKRKG---------
38299(HHH)      -----DPRKKLKELVDRAKTLAKQVGNKFILASVRILEELAKKAIDNNDEHLLELVEDQLEIAIDQLEY---
27200(EHEEHE)   ------SDVFFLVDSDEVAKSVKEIAKRYADRVYVQHHPG--HKVIILVRNVDPRIAEKIRKLFPQVDVTIH
```

d

TrkA

```
                        10        20        30        40        50        60
57445-evo       STQELVEELLELVRKDLEEARKLAKEIVKELGLSPELRFFLQLAIALAQRDPEVARRILERALNL
57445           STEELVEELLELARKDPQEARKLVEEIVKELGLSPERRFELQLAIVLAQRDPELARRILERALNH
```

IL6

```
                        10        20        30        40        50        60
25367-evo       GEEEVIEYLTREFKDDPELVRLLREAIEGLLKAGEDPEIVEMLIESLIHITGNPRVAVKLAKEYA
25367           GEEEVKEYLTRKFKDDPELVRLLREAIEVLLKAGEDPELVLSLIESLIHITGDPRAAVKLAKEFG
```

Fig. S6 | Sequence alignments for the most enriched binders.

Sequence alignments of the enriched binders identified from yeast display for D-Pep-1 (**a**), D-TrkA (**b**), and D-IL-6 (**c**). The protein topologies are show in parentheses (HHH, HHHH,

EHEEHE; where H indicates an α-helix and E a β-strand). **d**. Sequence alignments between the evolved binders and the initially designed binders.
