# Supplementary material for: Accurate de novo design of heterochiral protein–protein interactions
Source: Cell Res. 2024 Aug 14;34(12):846–58. doi: 10.1038/s41422-024-01014-2 (PMC11614891; doi:10.1038/s41422-024-01014-2)
Supplement: Supplementary file 7 — Supplementary information, Fig. S7 [file 41422_2024_1014_MOESM7_ESM.pdf]

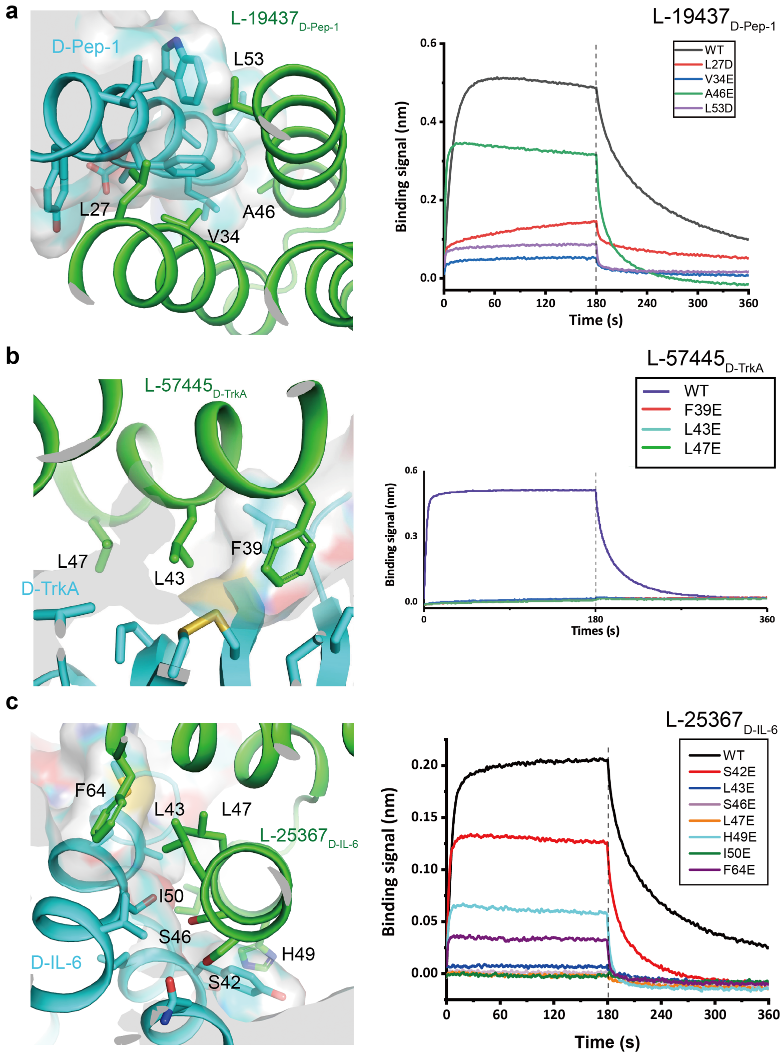

Fig. S7 | Validation of the designer protein-protein interface by mutagenesis and competition assays.

(**a**, **b** and **c**) Designer interface residues of L-protein binders are important for the binding to the D-targets. Interaction of the L-protein binder variants bound with D-targets were examined by biolayer interferometry (L-19437$_{\text{D-Pep-1}}$ (**a**), L-57445$_{\text{D-TrkA}}$ (**b**) and L-25367$_{\text{D-IL-6}}$ (**c**)). Left panel, zoom-in view of the designed interface. Key interface residues are highlighted in sticks. Right panel, biolayer interferometry results for the designer L-protein binder sequences (WT) and variants. Mutations of the interface residues reduced or totally abolished the binding signal.
