# Supplementary material for: Accurate de novo design of heterochiral protein–protein interactions
Source: Cell Res. 2024 Aug 14;34(12):846–58. doi: 10.1038/s41422-024-01014-2 (PMC11614891; doi:10.1038/s41422-024-01014-2)
Supplement: Supplementary file 8 — Supplementary information, Fig. S8 [file 41422_2024_1014_MOESM8_ESM.pdf]

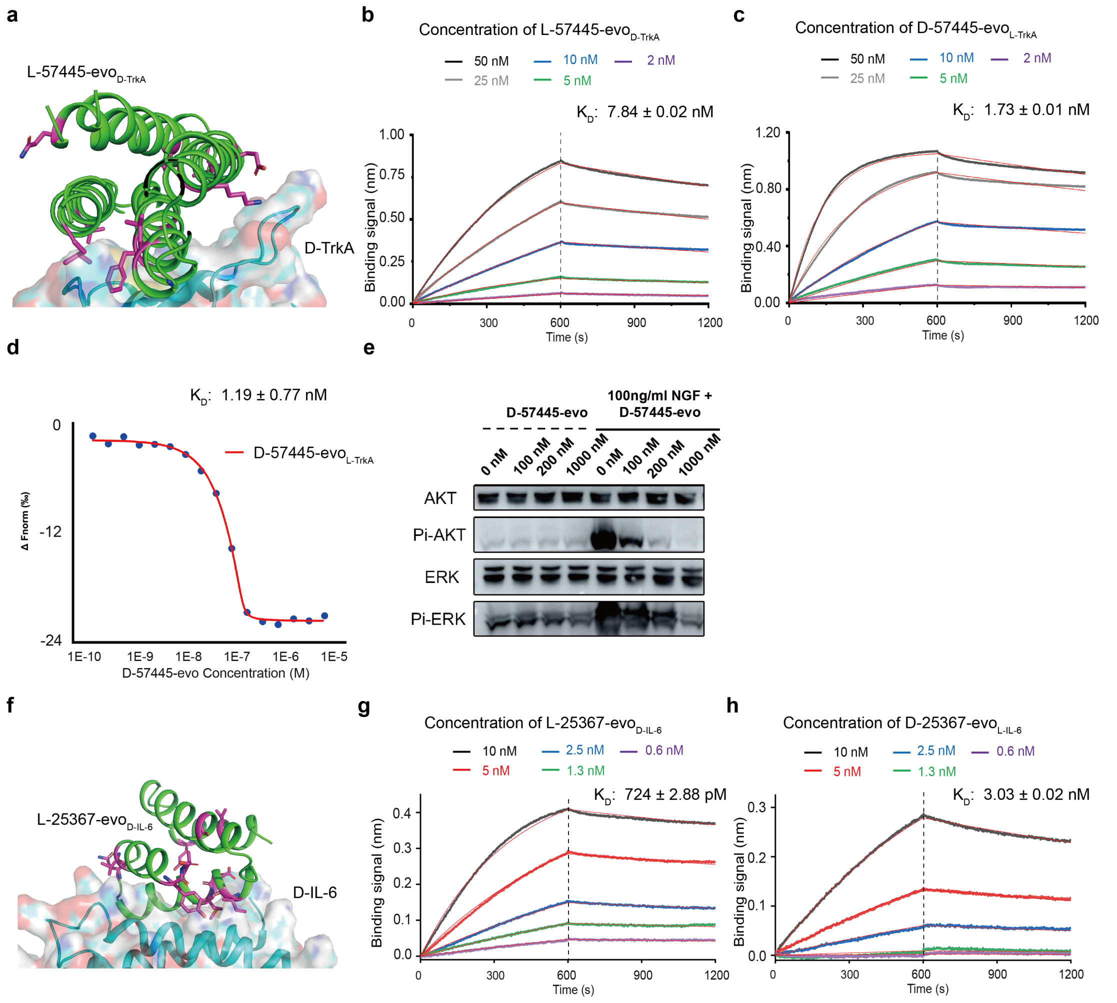

Fig. S8 | Characterization of evolved L-57445-evo$_{D\text{-}TrkA}$ and L-25367-evo$_{D\text{-}IL\text{-}6}$.

a, Computational design model of the complex between D-TrkA (cyan) and L-57445-evo$_{D\text{-}TrkA}$ (green), with the mutated positions colored magenta. **b,** Analysis of L-57445-evo$_{D\text{-}TrkA}$ binding with D-TrkA by using biolayer interferometry. **c,** Analysis of D-57445-evo$_{L\text{-}TrkA}$ binding with L-TrkA by using biolayer interferometry. **d,** Analysis of D-57445-evo$_{L\text{-}TrkA}$ binding with L-TrkA by using MST. Please refer to Table S5 for the fitted parameters. **e,** The effect of D-57445-evo$_{L\text{-}TrkA}$ on the phosphorylation of Akt and Erk kinases downstream of TrkA signaling was investigated. The phosphorylated forms of Akt and Erk kinases were indicated as Pi-AKT and Pi-ERK, respectively. The concentration of the D-57445-evo$_{L\text{-}TrkA}$ binder added to the experiment was

represented on the top of the gel. The left side of the gel corresponds to the condition without the addition of NGF, while the right side corresponds to the presence of 100 ng/ml NGF. **f,** Computational design model of the complex between D-IL-6 (cyan) and L-25367-evo$_{D\text{-}IL\text{-}6}$ (green) with the mutated positions colored magenta. **g,** Binding analysis of the L-25367-evo$_{D\text{-}IL\text{-}6}$ with D-IL-6 by using biolayer interferometry. **h,** Binding analysis of the D-25367-evo$_{L\text{-}IL\text{-}6}$ with L-IL-6 by using biolayer interferometry. Please refer to Table S2-4for the fitted parameters.
