# Supplementary material for: Accurate de novo design of heterochiral protein–protein interactions
Source: Cell Res. 2024 Aug 14;34(12):846–58. doi: 10.1038/s41422-024-01014-2 (PMC11614891; doi:10.1038/s41422-024-01014-2)
Supplement: Supplementary file 9 — Supplementary information, Fig. S9 [file 41422_2024_1014_MOESM9_ESM.pdf]

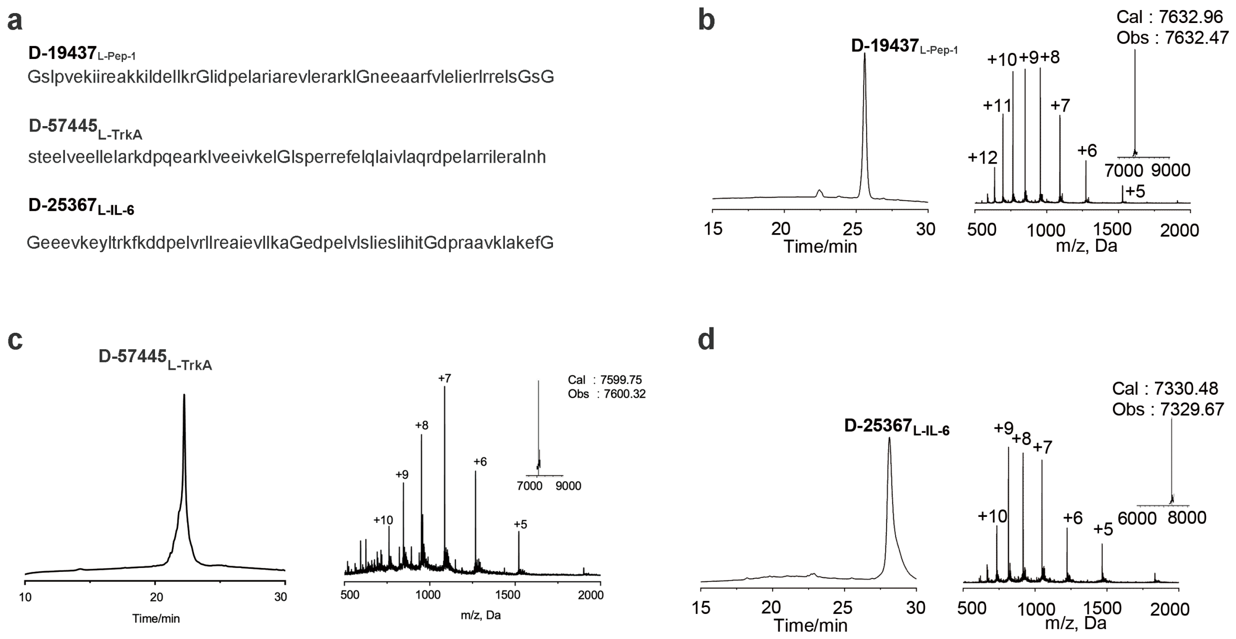

Fig. S9 | Chemical protein synthesis and characterization of D-protein binders.
a, Amino acid sequences of D-19437$_{\text{L-Pep-1}}$, D-57445$_{\text{L-TrkA}}$ and D-25367$_{\text{L-IL-6}}$. **b,** HPLC analysis and ESI-MS results of purified D-19437$_{\text{L-Pep-1}}$. **c,** HPLC analysis and ESI-MS results of purified D-57445$_{\text{L-TrkA}}$. **d,** HPLC analysis and ESI-MS results of purified D-25367$_{\text{L-IL-6}}$.
