# Supplementary material for: Accurate de novo design of heterochiral protein–protein interactions
Source: Cell Res. 2024 Aug 14;34(12):846–58. doi: 10.1038/s41422-024-01014-2 (PMC11614891; doi:10.1038/s41422-024-01014-2)
Supplement: Supplementary file 10 — Supplementary information, Fig. S10 [file 41422_2024_1014_MOESM10_ESM.pdf]

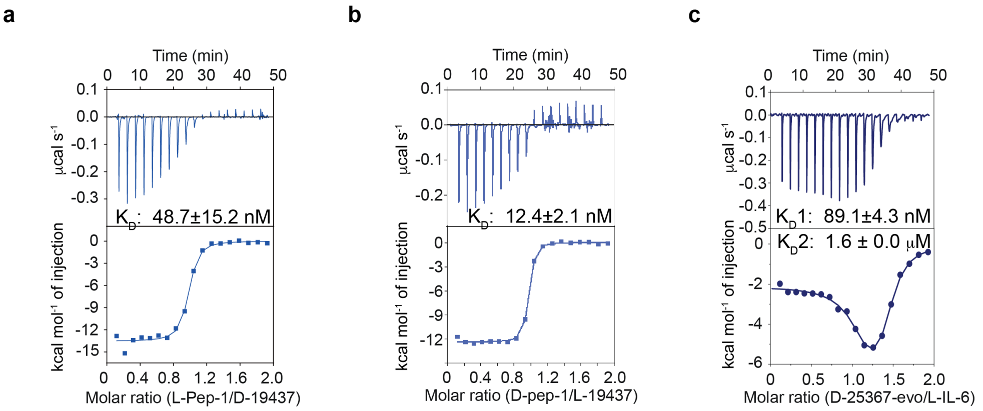

d

Name	N (sites)	K_D (nM)	ΔH (Kcal/mol)	ΔG (Kcal/mol)	-TΔS (Kcal/mol)
L-Pep-1/D-19437	0.932 ± 0.003	12.4 ± 2.1	-13.5 ± 0.34	-9.99	1.59
D-Pep-1/L-19437	0.938 ± 0.010	48.7 ± 15.2	-12.4 ± 0.12	-10.80	3.56
D-25367-evo/L-IL-6	1.040 ± 0.009	89.1 ± 4.3	-2.10 ± 0.06	-9.62	-7.52
D-25367-evo/L-IL-6-2	0.388 ± 0.012	1580.0 ± 69.2	-8.34 ± 0.24	-7.91	0.43

Fig. S10. Interaction analysis of the binders bound with targets by using isothermal titration calorimetry.

a, L-Pep-1 was titrated over D-19437. **b,** D-Pep-1 was titrated over L-19437. **c,** D-25367-evo was titrated over L-IL-6. **d,** ITC measurement of the interactions. For D-25367-evo, the ITC analysis suggested the possible presence of two sequential D-protein binding sites on L-IL-6 (D-25367-evo/L-IL-6 and D-25367-evo/L-IL-6-2).
