# Supplementary material for: Accurate de novo design of heterochiral protein–protein interactions
Source: Cell Res. 2024 Aug 14;34(12):846–58. doi: 10.1038/s41422-024-01014-2 (PMC11614891; doi:10.1038/s41422-024-01014-2)
Supplement: Supplementary file 11 — Supplementary information, Fig. S11 [file 41422_2024_1014_MOESM11_ESM.pdf]

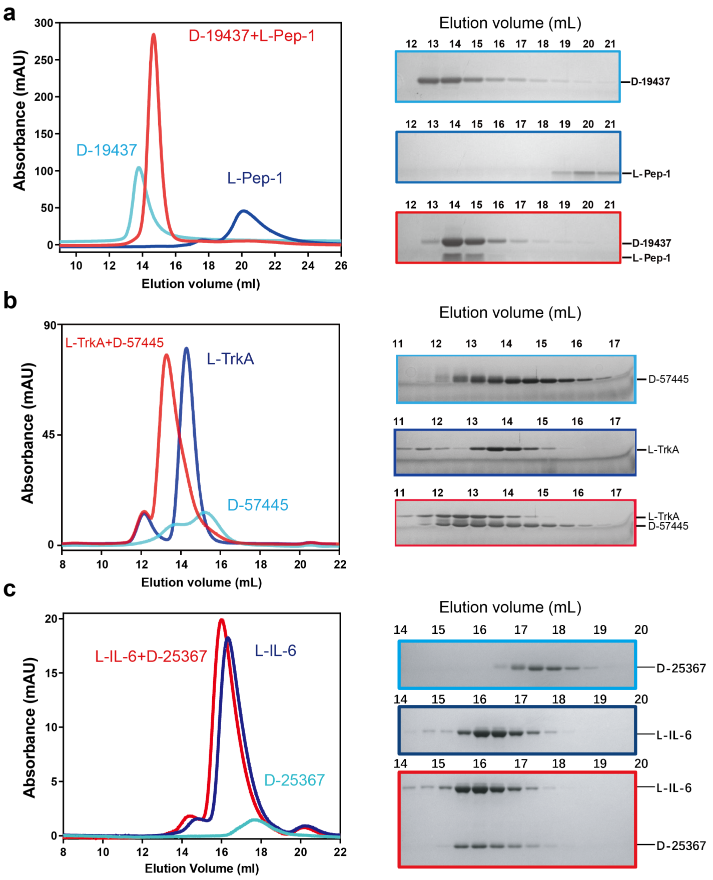

Fig. S11 | The designer D-protein binders co-migrated with the cognate L-protein target during size exclusion chromatography (SEC).

SEC analyses of the individual components and complexes of D-19437/L-Pep-1 (**a**), D-57445/L-TrkA (**b**) and D-25367/L-IL-6 (**c**). SEC eluent fractions were applied to SDS-PAGE and stained by Coomassie-blue. D-19437 is likely to exist as a dimeric form in solution. However, in the presence of L-Pep-1, the D-19437 dimer dissociates, leading to the formation of the D-19437/L-Pep-1 complex. As a result, the elution of the D-19437 species occurs prior to that of the D-19437/L-Pep-1 complex.
