# Supplementary material for: Accurate de novo design of heterochiral protein–protein interactions
Source: Cell Res. 2024 Aug 14;34(12):846–58. doi: 10.1038/s41422-024-01014-2 (PMC11614891; doi:10.1038/s41422-024-01014-2)
Supplement: Supplementary file 12 — Supplementary information, Fig. S12 [file 41422_2024_1014_MOESM12_ESM.pdf]

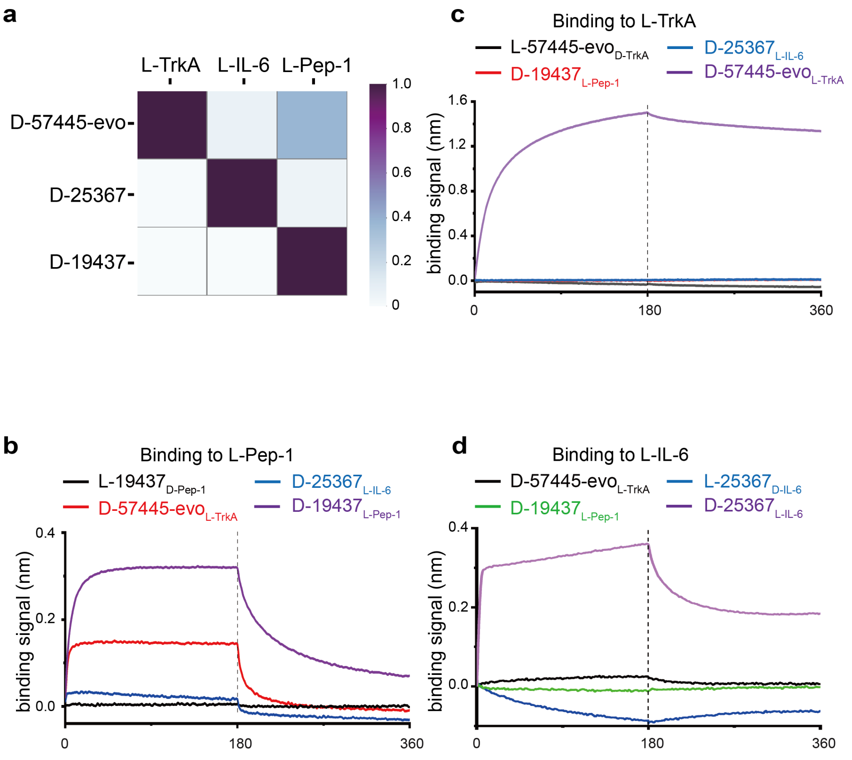

Fig. S12 | High enantiomeric and target specificity for the designer D-protein binders. **a,** Normalized maximal biolayer interferometry response signal for each D-protein binder and naturally existing target pair was shown in the heat map. (**b-d).** Biolayer interferometry analysis of the L- and D-protein binders bound to L-Pep-1 (**b**), L-TrkA (**c**) and L-IL-6 (**d**).
