# Supplementary material for: Accurate de novo design of heterochiral protein–protein interactions
Source: Cell Res. 2024 Aug 14;34(12):846–58. doi: 10.1038/s41422-024-01014-2 (PMC11614891; doi:10.1038/s41422-024-01014-2)
Supplement: Supplementary file 13 — Supplementary information, Fig. S13 [file 41422_2024_1014_MOESM13_ESM.pdf]

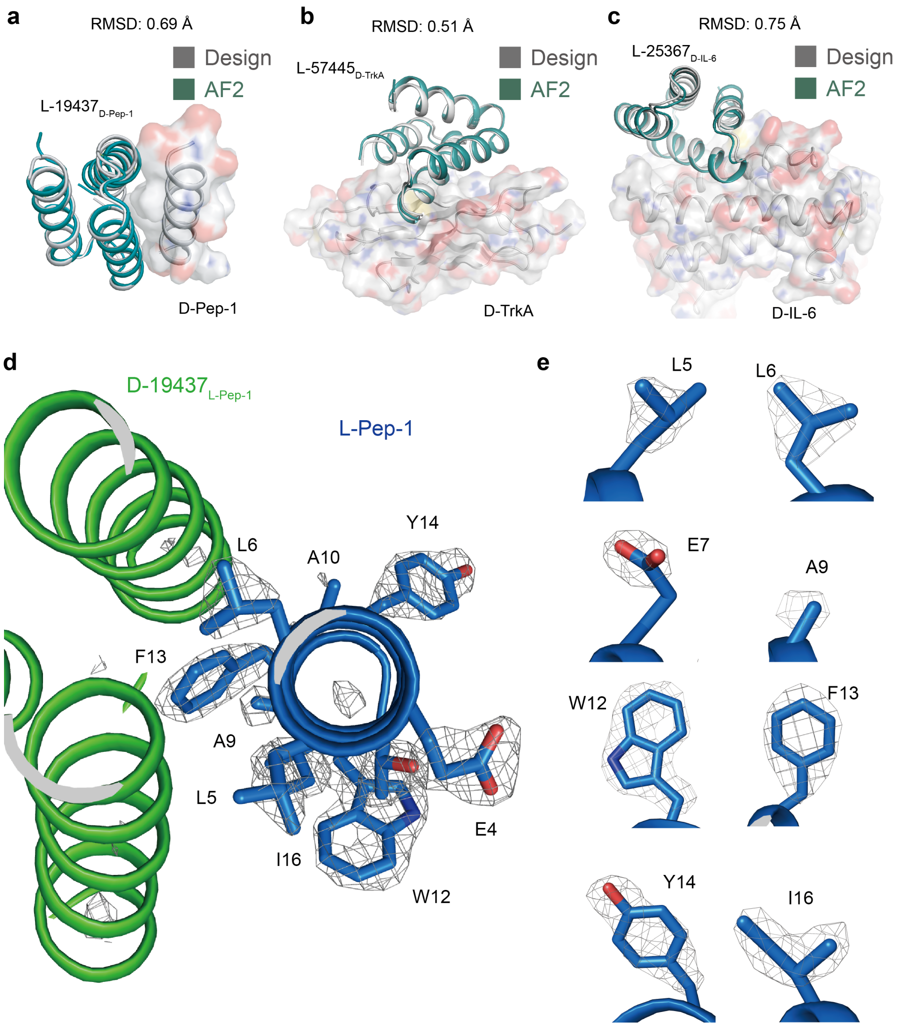

Fig. S13 | Structural validation.

(**a**, **b** and **c**) Prediction of 3D structures of the L-protein binders by using AlphaFold2. Predicted L-protein binder structures (dark cyan) were superimposed to the cognate design models (gray) of the L-19437/D-Pep-1 (**a**), L-57445/D-TrkA (**b**) and L-25367/D-IL-6 (**c**) complexes. D-targets are shown in surface and cartoon representation. (**d** and **e**) Unbiased $F_{\text{obs}} - F_{\text{calc}}$ (gray mesh, contoured at 3σ) electron-density maps for L-Pep-1. **d,** Additional density was observed for all the residues at the interface with D-19437$_{\text{L-Pep-1}}$ and most of the large side chains of L-Pep-1 in an unbiased $F_{\text{obs}} - F_{\text{calc}}$ map. The polder map[63] was calculated by using a complex structure model with residues 4-10, 12-14 and 16-17 of L-Pep-1 truncated to glycine. D-19437$_{\text{L-Pep-1}}$ is

in green; L-Pep-1 is in blue. **e,** Zoomed-in view of polder map for representative interface residues and bulky residues of L-Pep-1.
