# Supplementary material for: Accurate de novo design of heterochiral protein–protein interactions
Source: Cell Res. 2024 Aug 14;34(12):846–58. doi: 10.1038/s41422-024-01014-2 (PMC11614891; doi:10.1038/s41422-024-01014-2)
Supplement: Supplementary file 14 — Supplementary information, Fig. S14 [file 41422_2024_1014_MOESM14_ESM.pdf]

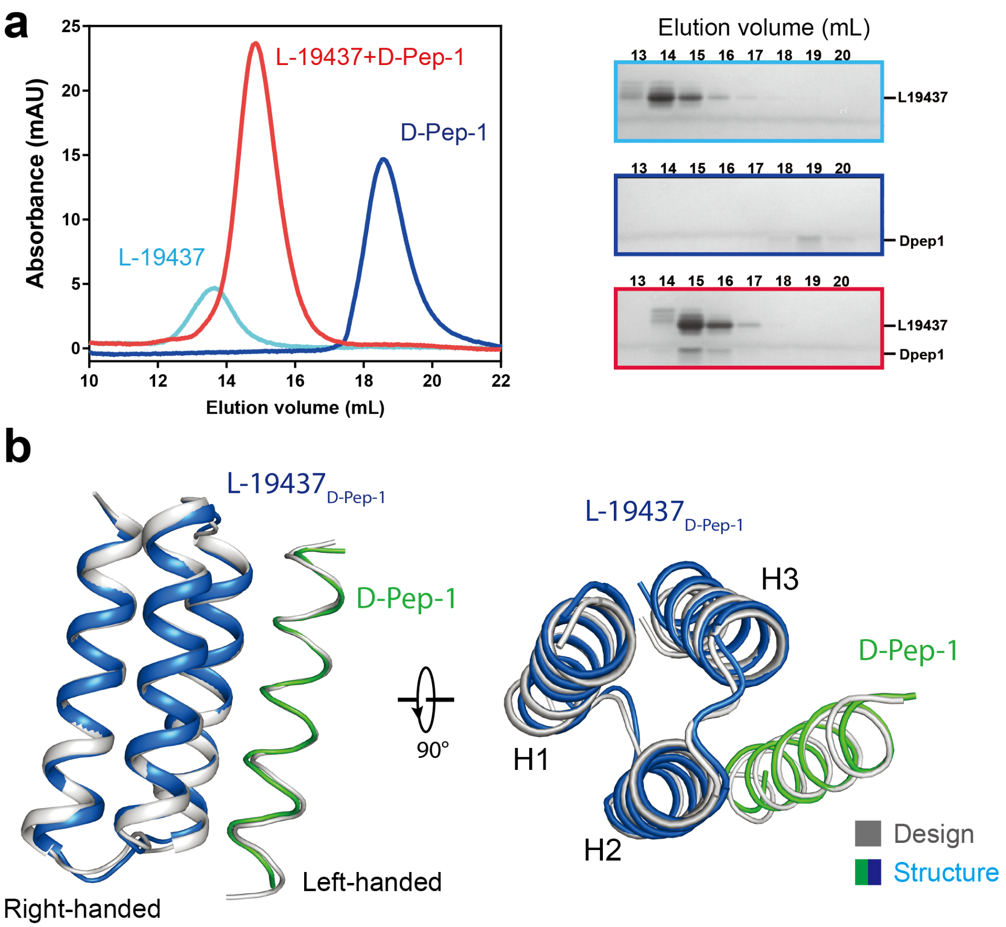

Fig. S14. Crystal structure of the L-19437-D-Pep-1 heterochiral protein complex agrees with the design model.

a, SEC analyses of the individual components and the complex of the L-19437/D-Pep-1. The designer binder L-19437 co-migrated with D-Pep-1 during size exclusion chromatography (SEC). SEC eluent fractions were applied to SDS-PAGE and stained by Coomassie-blue. **b**, Superposition of the crystal structure (D-Pep-1 in green; L-$19437_{\text{D-Pep-1}}$ in blue) and the design model (gray). The crystal structure is nearly identical to the design model, with a Cα RMSD value of 0.64 Å.
