# Supplementary material for: Accurate de novo design of heterochiral protein–protein interactions
Source: Cell Res. 2024 Aug 14;34(12):846–58. doi: 10.1038/s41422-024-01014-2 (PMC11614891; doi:10.1038/s41422-024-01014-2)
Supplement: Supplementary file 15 — Supplementary information, Fig. S15 [file 41422_2024_1014_MOESM15_ESM.pdf]

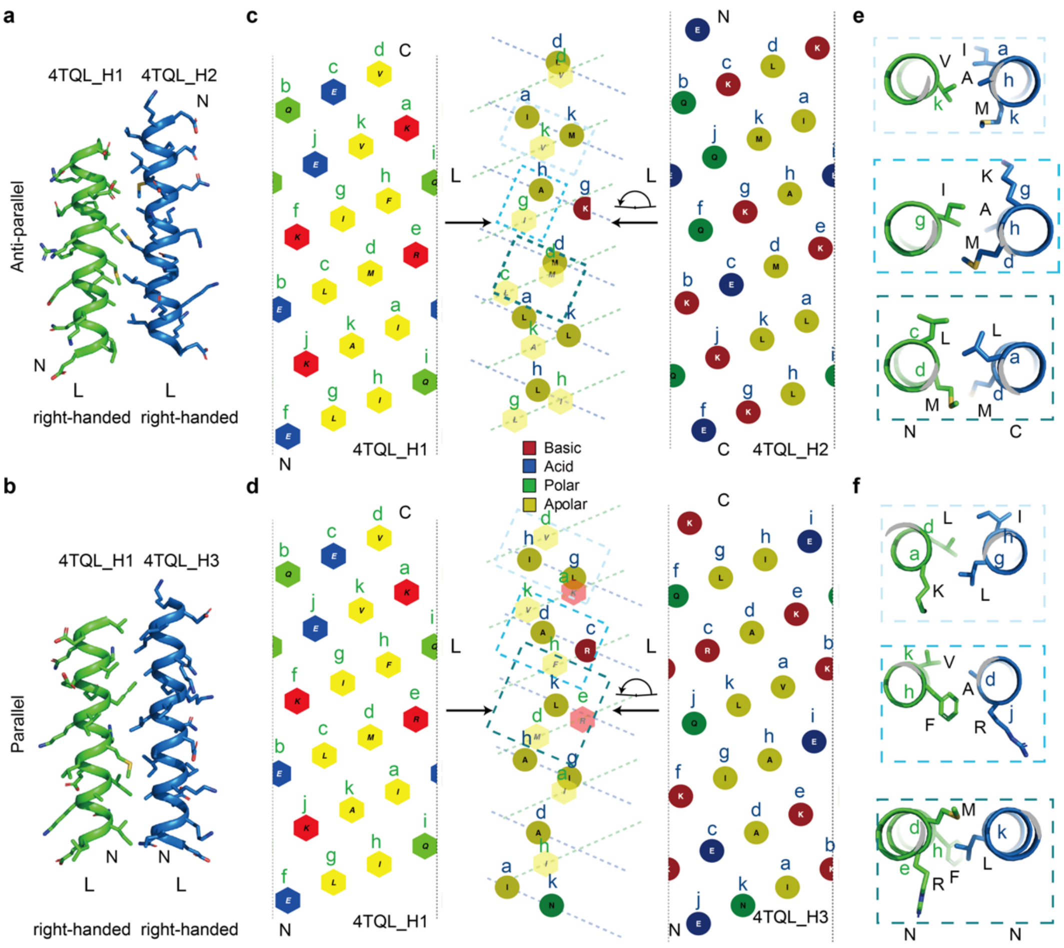

Fig. S15 | Homochiral helix-helix interactions in an untwisted 3-helix bundle.

In the heptad repeat model for the most common homochiral coiled-coils, helices are supercoiled and have periodicities of approximately 3.5 residues/turn[33]. Here we analyze a homochiral untwisted α-helix bundle (PDB code: 4TQL) comprising H1, H2, and H3 helices with periodicities of 3.6, 3.69, and 3.7 residues/turn, respectively, comparable to those for L-Pep-1, D-19437-H2, and D-19437-H3. (**a** and **b**) Homochiral helical interfaces between helices of an untwisted 3-helix bundle (PDB code: 4TQL). The first helix of 4TQL (4TQL_H1, residues 32–52) forms anti-parallel and parallel helix-helix interactions with the second (4TQL_H2, residues 109–131) and the third (4TQL_H3, residues 188–210) helices, respectively. 4TQL_H1 in green; 4TQL_H2 and 4TQL_H3 in blue. (**c** and **d**) Helical net diagrams. Left panels, 4TQL_H1. Right panels, 4TQL_H2

(**c**) and 4TQL_H3 (**d**). Middle panels, homochiral helix-helix interface. **c,** anti-parallel. **d,** parallel. The hexagons indicate the positions of Cα atoms for 4TQL_H1 (circles for 4TQL_H2 and 4TQL_H3). The scheme for display is similar to that in Fig. 5. (**e** and **f**) Homochiral helix-helix packing arrangements in each of the three packing layers. **e,** anti-parallel. **f,** parallel.
