# Supplementary material for: Accurate de novo design of heterochiral protein–protein interactions
Source: Cell Res. 2024 Aug 14;34(12):846–58. doi: 10.1038/s41422-024-01014-2 (PMC11614891; doi:10.1038/s41422-024-01014-2)
Supplement: Supplementary file 16 — Supplementary information, Table S1 [file 41422_2024_1014_MOESM16_ESM.pdf]

Table S1. Statistics of diffraction data collection and refinement.

The diffraction dataset for each complex was collected from a single crystal. Values in parentheses are for the highest resolution shell. $R_{merge}=\Sigma_h\Sigma_i|I_{h,i}-I_h|/\Sigma_h\Sigma_i I_{h,i}$, where I_h is the mean intensity of the i observations of symmetry related reflections of h. $R=\Sigma|F_{obs}-F_{calc}|/\Sigma F_{obs}$, where F_{calc} is the calculated protein structure factor from the atomic model (R_{free} was calculated with 5% of the reflections selected).

Data	D-19437-L-Pep-1	L-19437-D-Pep-1
Wavelength	1.54	1.54
Integration Package	HKL2000	HKL2000
Space Group	P 21 21 2	P 21 21 2
Unit Cell (Å)	53.40, 82.28, 35.32	54.06, 81.92, 35.72
Unit Cell (°)	90, 90, 90	90, 90, 90
Resolution (Å)<br>Outer shell (Å)	24.40 - 2.00 (2.07- 2.00)	24.37 - 2.20 (2.24- 2.20)
R_{merge}	0.067 (0.436)	0.055 (0.240)
I/sigma	15.44 (3.00)	14.12 (5.54)
$CC_{1/2}$	0.998 (0.975)	0.998 (0.975)
Completeness (%)	97.95 (82.76)	97.82 (87.37)
Number of unique reflections	10862 (894)	8413(733)
Redundancy	4.2 (3.1)	4.0 (3.5)
R_{work} /R_{free}	0.219/0.226	0.202/0.249
No. atoms		
Overall	1415	1440
Non-solvent	1335	1360
Solvent	80	80
Average B value (Å^2)	26.83	30.95
R.m.s. deviations		
Bonds (Å)	0.006	0.007
Angle (°)	0.89	0.80
Ramachandran plot statistics (%)		
Most favourable	100	100
Additionally allowed	0.0	0.0
Generously allowed	0.0	0.0
Disallowed	0.0	0.0
