# Supplementary material for: Accurate de novo design of heterochiral protein–protein interactions
Source: Cell Res. 2024 Aug 14;34(12):846–58. doi: 10.1038/s41422-024-01014-2 (PMC11614891; doi:10.1038/s41422-024-01014-2)
Supplement: Supplementary file 17 — Supplementary information, Table S2 [file 41422_2024_1014_MOESM17_ESM.pdf]

Table S2. Statistics of BLI data for Pep-1

	KD (M)	KD Error	ka (1/Ms)	ka Error	kdis (1/s)	kdis Error	Full R^2
L-Pep-1/D-19437-1	5.96E-08	9.80E-10	2.72E+05	4.28E+03	1.62E-02	7.97E-05	0.9913
L-Pep-1/D-19437-2	3.76E-08	3.30E-10	3.24E+05	2.73E+03	1.22E-02	3.01E-05	0.9791
D-Pep-1/L-19437-1	2.27E-08	3.00E-10	2.73E+05	3.73E+03	6.20E-03	2.79E-05	0.9811
D-Pep-1/L-19437-2	3.15E-08	3.74E-10	3.38E+05	3.91E+03	1.07E-02	3.01E-05	0.9683
