# Supplementary material for: Accurate de novo design of heterochiral protein–protein interactions
Source: Cell Res. 2024 Aug 14;34(12):846–58. doi: 10.1038/s41422-024-01014-2 (PMC11614891; doi:10.1038/s41422-024-01014-2)
Supplement: Supplementary file 18 — Supplementary information, Table S3 [file 41422_2024_1014_MOESM18_ESM.pdf]

Table S3. Statistics of BLI data for TrkA.

	KD (M)	KD Error	ka (1/Ms)	ka Error	kdis (1/s)	kdis Error	Full R^2
D-TrkA/L-57445	1.52E-07	1.58E-09	3.34E+05	3.28E+03	5.08E-02	1.65E-04	0.9957
L-TrkA/D-57445	1.51E-07	2.28E-09	2.32E+05	3.32E+03	3.51E-02	1.65E-04	0.9857
D-TrkA/L-57445-evo-1	7.85E-09	1.90E-11	3.78E+04	6.57E+01	2.97E-04	4.98E-07	0.9995
D-TrkA/L-57445-evo-2	1.09E-08	5.17E-11	2.94E+04	7.10E+01	3.22E-04	1.31E-06	0.9935
L-TrkA/D-57445-evo-1	1.73E-09	5.52E-12	1.44E+05	1.78E+02	2.48E-04	7.31E-07	0.9986
L-TrkA/D-57445-evo-2	1.80E-09	6.57E-12	1.35E+05	1.87E+02	2.43E-04	8.17E-07	0.9927
