# Supplementary material for: Accurate de novo design of heterochiral protein–protein interactions
Source: Cell Res. 2024 Aug 14;34(12):846–58. doi: 10.1038/s41422-024-01014-2 (PMC11614891; doi:10.1038/s41422-024-01014-2)
Supplement: Supplementary file 19 — Supplementary information, Table S4 [file 41422_2024_1014_MOESM19_ESM.pdf]

Table S4. Statistics of BLI data for IL-6.

	KD (M)	KD Error	ka (1/Ms)	ka Error	kdis (1/s)	kdis Error	Full R^2
D-IL6/L-25367-1	2.51E-08	2.27E-10	3.02E+05	2.55E+03	7.58E-03	2.46E-05	0.9725
D-IL6/L-25367-2	1.18E-08	1.46E-10	6.44E+05	7.45E+03	7.58E-03	3.45E-05	0.9524
L-IL6/D-25367-1	2.83E-08	2.85E-10	3.91E+05	3.73E+03	1.11E-02	3.64E-05	0.9701
L-IL6/D-25367-2	4.83E-08	4.19E-10	2.11E+05	1.73E+03	1.02E-02	2.86E-05	0.9829
L-IL-6/D-25367-evo-1	3.03E-09	1.70E-11	1.08E+05	5.23E+02	3.28E-04	9.33E-07	0.9987
L-IL-6/D-25367-evo-2	8.75E-09	3.68E-10	2.28E+04	9.35E+02	1.99E-04	1.80E-06	0.9965
D-IL-6/L-25367-evo-1	7.24E-10	2.88E-12	2.56E+05	4.65E+02	1.85E-04	6.54E-07	0.9989
D-IL-6/L-25367-evo-2	1.68E-09	1.20E-11	2.75E+05	1.46E+03	4.60E-04	2.19E-06	0.9919
