# Supplementary material for: Accurate de novo design of heterochiral protein–protein interactions
Source: Cell Res. 2024 Aug 14;34(12):846–58. doi: 10.1038/s41422-024-01014-2 (PMC11614891; doi:10.1038/s41422-024-01014-2)
Supplement: Supplementary file 20 — Supplementary information, Table S5 [file 41422_2024_1014_MOESM20_ESM.pdf]

Table S5. Statistics of MST data for TrkA.

	Fit Model	Bound	Unbound	KD	TargetConc	Standard Deviation	KD Confidence
L-TrkA/D-57445-evo	KD	917.9723	939.4028	1.19E-09	1.01E-07	0.380914	±7.7E-10
